# BCL7A’s arginine anchor links nucleosome recognition to chromatin remodeling and diffuse large B-cell lymphoma tumor suppression

**DOI:** 10.1093/procel/pwaf114

**Published:** 2026-01-02

**Authors:** Jingdong Xue, Kai Tian, Xiang Xu, Yuqian Feng, Ming Yu, Min Hao, Mingqian Hu, Wenhan Wang, Jiao Ma, Yixuan Pan, Mengyuan Peng, Jun Wu, Shuang He, Xizi Chen, Yanhui Xu, Wenjuan Wang, Yimin Lao, Bing Li

**Affiliations:** Department of Biochemistry and Molecular Cell Biology, Key Laboratory of Cell Differentiation and Apoptosis of Chinese Ministry of Education, Shanghai Key Laboratory for Tumor Microenvironment and Inflammation, Shanghai Jiao Tong University School of Medicine, Shanghai 200025, China; Department of Orthopedic Oncology, Shanghai Bone Tumor Institute, Shanghai General Hospital, Shanghai Jiao Tong University School of Medicine, Shanghai 200025, China; Department of Laboratory Medicine, Jiading Branch of Shanghai General Hospital, Shanghai Jiao Tong University School of Medicine, Shanghai 200025, China; Department of Biochemistry and Molecular Cell Biology, Key Laboratory of Cell Differentiation and Apoptosis of Chinese Ministry of Education, Shanghai Key Laboratory for Tumor Microenvironment and Inflammation, Shanghai Jiao Tong University School of Medicine, Shanghai 200025, China; Department of Biochemistry and Molecular Cell Biology, Key Laboratory of Cell Differentiation and Apoptosis of Chinese Ministry of Education, Shanghai Key Laboratory for Tumor Microenvironment and Inflammation, Shanghai Jiao Tong University School of Medicine, Shanghai 200025, China; Department of Biochemistry and Molecular Cell Biology, Key Laboratory of Cell Differentiation and Apoptosis of Chinese Ministry of Education, Shanghai Key Laboratory for Tumor Microenvironment and Inflammation, Shanghai Jiao Tong University School of Medicine, Shanghai 200025, China; Department of Biochemistry and Molecular Cell Biology, Key Laboratory of Cell Differentiation and Apoptosis of Chinese Ministry of Education, Shanghai Key Laboratory for Tumor Microenvironment and Inflammation, Shanghai Jiao Tong University School of Medicine, Shanghai 200025, China; Department of Biochemistry and Molecular Cell Biology, Shanghai Jiao Tong University School of Medicine, Shanghai 200025, China; Department of Biochemistry and Molecular Cell Biology, Shanghai Jiao Tong University School of Medicine, Shanghai 200025, China; Department of Biochemistry and Molecular Cell Biology, Key Laboratory of Cell Differentiation and Apoptosis of Chinese Ministry of Education, Shanghai Key Laboratory for Tumor Microenvironment and Inflammation, Shanghai Jiao Tong University School of Medicine, Shanghai 200025, China; Department of Biochemistry and Molecular Cell Biology, Key Laboratory of Cell Differentiation and Apoptosis of Chinese Ministry of Education, Shanghai Key Laboratory for Tumor Microenvironment and Inflammation, Shanghai Jiao Tong University School of Medicine, Shanghai 200025, China; Department of Laboratory Medicine, Jiading Branch of Shanghai General Hospital, Shanghai Jiao Tong University School of Medicine, Shanghai 200025, China; Clinicopathological Diagnosis & Research Center, The Affiliated Hospital of Youjiang Medical University for Nationalities, Baise 533000, China; Key Laboratory of Tumor Molecular Pathology of Guangxi Higher Education Institutes, Baise 533000, China; Fudan University Shanghai Cancer Center, Institutes of Biomedical Sciences, New Cornerstone Science Laboratory, State Key Laboratory of Genetics and Development of Complex Phenotypes, Department of Biochemistry and Biophysics, School of Life Sciences, Shanghai Key Laboratory of Radiation Oncology and Shanghai Key Laboratory of Medical Epigenetics, Shanghai Medical College of Fudan University, Shanghai 200032, China; Fudan University Shanghai Cancer Center, Institutes of Biomedical Sciences, New Cornerstone Science Laboratory, State Key Laboratory of Genetics and Development of Complex Phenotypes, Department of Biochemistry and Biophysics, School of Life Sciences, Shanghai Key Laboratory of Radiation Oncology and Shanghai Key Laboratory of Medical Epigenetics, Shanghai Medical College of Fudan University, Shanghai 200032, China; Fudan University Shanghai Cancer Center, Institutes of Biomedical Sciences, New Cornerstone Science Laboratory, State Key Laboratory of Genetics and Development of Complex Phenotypes, Department of Biochemistry and Biophysics, School of Life Sciences, Shanghai Key Laboratory of Radiation Oncology and Shanghai Key Laboratory of Medical Epigenetics, Shanghai Medical College of Fudan University, Shanghai 200032, China; Reproduction Medical Center, Xinhua Hospital Affiliated to Shanghai Jiao Tong University School of Medicine, Shanghai 200025, China; Department of Biochemistry and Molecular Cell Biology, Key Laboratory of Cell Differentiation and Apoptosis of Chinese Ministry of Education, Shanghai Key Laboratory for Tumor Microenvironment and Inflammation, Shanghai Jiao Tong University School of Medicine, Shanghai 200025, China; Department of Biochemistry and Molecular Cell Biology, Key Laboratory of Cell Differentiation and Apoptosis of Chinese Ministry of Education, Shanghai Key Laboratory for Tumor Microenvironment and Inflammation, Shanghai Jiao Tong University School of Medicine, Shanghai 200025, China

**Keywords:** hSWI/SNF, chromatin remodeling, arginine anchor, DLBCL

## Abstract

Dysfunction of human chromatin remodeling complex switch/sucrose non-fermentable (hSWI/SNF) associates with multiple diseases including cancer. BCL7A, a tissue-specific, non-catalytic subunit of this complex, exhibits tumor-­suppressive activity, especially in diffuse large B-cell lymphoma (DLBCL). However, the underlying mechanism remains elusive. In this study, we use protein structural prediction to identify a conserved arginine anchor in the N-terminal α-helix of BCL7A and demonstrate that this arginine anchor is crucial for the chromatin remodeling activity of the hSWI/SNF complexes. Truncation or DLBCL-associated mutations of this anchor impair BCL7A’s tumor-suppressive function without affecting its integration into the complexes. Instead, these mutations lead to decreased BCL7A occupancy at target loci and reduced chromatin accessibility and transcriptional regulation. *In vivo* and cellular assays further validate the pivotal role of the arginine anchor in BCL7A-mediated tumor suppression. Mechanistically, we reveal that BCL7A regulates histone displacement, and its arginine anchor and the SMARCB1 subunit work cooperatively to regulate the remodeling activity of canonical BAF (BRG1/BRM-associated factor) complexes. Altogether, our study identifies the BCL7A arginine anchor as a key molecular switch that links nucleosome binding to chromatin remodeling and tumor suppression, making it a potential therapeutic target for DLBCL.

## Introduction

Human switch/sucrose non-fermentable (hSWI/SNF) chromatin remodeling complexes, also known as BAF (BRG1/BRM-associated factor), are crucial for regulating chromatin accessibility in an ATP-dependent manner. They regulate differentiation, development, and disease pathogenesis (Becker and Workman, 2013; Bieluszewski et al., 2023; Gourisankar et al., 2024). The family includes canon­ical BAF (cBAF), polybromo-associated BAF (PBAF), and non-canonical BAF (ncBAF). Each variant consists of a core ATPases (BRG1 or BRM), multiple structural subunits (BAF155 and BAF170), and a variety of auxiliary paralogous subunits that provide specific temporal and spatial control upon transcriptional activation (Mashtalir et al., 2018; Schick et al., 2019). The three hSWI/SNF complexes exhibit distinct genomic localization patterns, reflecting their functional divergence. Specifically, cBAF and PBAF preferentially associate with enhancers and promoters, respectively, where they facilitate DNA accessibility to enable transcriptional regulation. ncBAF localizes to promoters and sites occupied by the architectural protein CTCF, implying a potential dual role in modulating promoter accessibility and mediating chromatin loop formation (Bieluszewski et al., 2023). Intriguingly, about 20% of human cancers correlate with the mutation of one or more of the 30 genes encoding the components of the hSWI/SNF complex, highlighting its significant role in tumorigenesis and potential as therapeutic target (Kadoch et al., 2013).

The nucleosome acidic patch, primarily composed of negatively charged residues from the H2A/H2B dimer, is pivotal for the binding of chromatin remodeling complexes, which often utilize arginine anchors for interaction (Li et al., 2007; Mariño-Ramírez et al., 2005; Paul, 2021). This site specifically attracts the arginine-rich motifs of various chromatin-binding complexes, including ISWI, SAGA, and NuA4, facilitating their remodeling activities (Dao et al., 2020; Makde et al., 2010; Morgan et al., 2016; Pathare et al., 2020; Qu et al., 2022). In the context of human SWI/SNF complexes, the SnAc domain of SMARCA4 and the arginine-rich C-terminal domain (CTD) of SMARCB1 engage with the acidic patch, albeit on different sides of the nucleosome, to mediate remodeling functions (He et al., 2020; Mashtalir et al., 2020; Sen et al., 2013; Yuan et al., 2022). Notably, in synovial sarcoma, the SS18-SSX fusion protein introduces an aberrant arginine anchor via the SSX component, which disrupts normal acidic patch interactions and compromises chromatin remodeling, thereby contributing to oncogenesis (McBride et al., 2018, 2020). The multiplicity of arginine anchors in hSWI/SNF complexes suggests a potential for diverse conformational states during nucleosome interaction, although the specific mechanisms underlying these dynamic engagements remain to be elucidated.

The BCL7 family, comprising BCL7A, BCL7B, and BCL7C, functions as a monomer within the cBAF, PBAF, and ncBAF complexes, integrating into the ARP (actin-related protein) module alongside ACTL6A/B and ACTB, and interacting with the HSA (Helicase-SANT-associated) domain of SMARCA2/4 (Candela-Ferre et al., 2024; Schubert et al., 2013).The ARP module, a conserved component of SWI/SNF complexes across eukaryote, plays a crucial role in complex integrity, subcellular localization, and chromatin remodeling activity to regulate gene expression and maintain genome integrity (Cairns et al., 1998; Mashtalir et al., 2018; Szerlong et al., 2003; Tea and Luo, 2011; Wu et al., 2007; Zhao et al., 1998). Markedly, dysregulation of ARP module components, especially ACTL6A/B, correlates with tumorigenesis of squamous cell carcinoma and gastric cancer (Chang et al., 2021; Saladi et al., 2017; Yang et al., 2023).

Emerging evidence highlights BCL7A as a multifunctional regulator with critical roles in tumor suppression, clinical prognosis, and neural development. As a potential prognostic and predictive marker, BCL7A demonstrates significant clinical relevance across malignancies. In ovarian cancer, low BCL7A expression independently associates with reduced overall and relapse-free survival (Sun et al., 2019), while in glioma, decreased BCL7A levels correlate with higher tumor grade and predict poorer outcomes for patients undergoing combined chemoradiotherapy (Liu et al., 2021). The tumor-suppressive functions of BCL7A are mediated through diverse molecular mechanisms. In diffuse large B-cell lymphoma (DLBCL), biallelic inactivation of BCL7A disrupts its integration into the SWI/SNF chromatin remodeling complex, impairing tumor suppression, whereas its restoration reactivates antitumor activity through B-cell pathways (Baliñas-Gavira et al., 2020). Similarly, in acute myeloid leukemia (AML), BCL7A silencing via promoter methylation promotes tumor progression, and its re-expression inhibits growth while enhancing chemosensitivity through the IRF7/HMGCS1 axis (Li et al., 2024; Patiño-Mercau et al., 2023). Multiple myeloma studies further reveal that BCL7A loss, caused by noncoding mutations, compromises its ability to inhibit IRF4-mediated oncogenic activity (Chakraborty et al., 2025). Beyond its oncological significance, BCL7A plays distinct roles in neural development, regulating Notch/Wnt signaling and mitochondrial bioenergetics—functions not shared by its homolog BCL7B (Wischhof et al., 2017, 2022). These diverse roles position BCL7A as a pleiotropic factor whose complete molecular mechanisms, particularly in tumor suppression, warrant further investigation. However, as a regulatory subunit of chromatin remodeling complexes, the detailed epigenetic molecular mechanisms underlying these effects remain largely unknown.

We aim to elucidate the hSWI/SNF modulation and tumor-suppressive activity of BCL7A. Using structural analysis, we identify an N-terminal α-helix of BCL7A, which is rich in lysine and arginine residues, that may interact with nucleosome acidic patch and regulates hSWI/SNF chromatin remodeling activity. Next, we employ both *in vitro* and *in vivo* functional analyses to demonstrate that BCL7A engages with the nucleosome acidic patch through a conserved arginine anchor. This interaction, in coordination with SMARCB1, regulates hSWI/SNF activity, particularly histone displacement. Notably, somatic mutations in the arginine anchor of BCL7A that disrupt the nucleosome interaction correlate with suppressed proliferation in DLBCL cells. Together, our results reveal a novel mechanism by which BCL7A regulates chromatin accessibility and underscore its role in the pathogenesis of DLBCL.

## Results

### BCL7A employs distinct structural domains for hSWI/SNF complex incorporation and nucleosome binding

**Figure 1.**
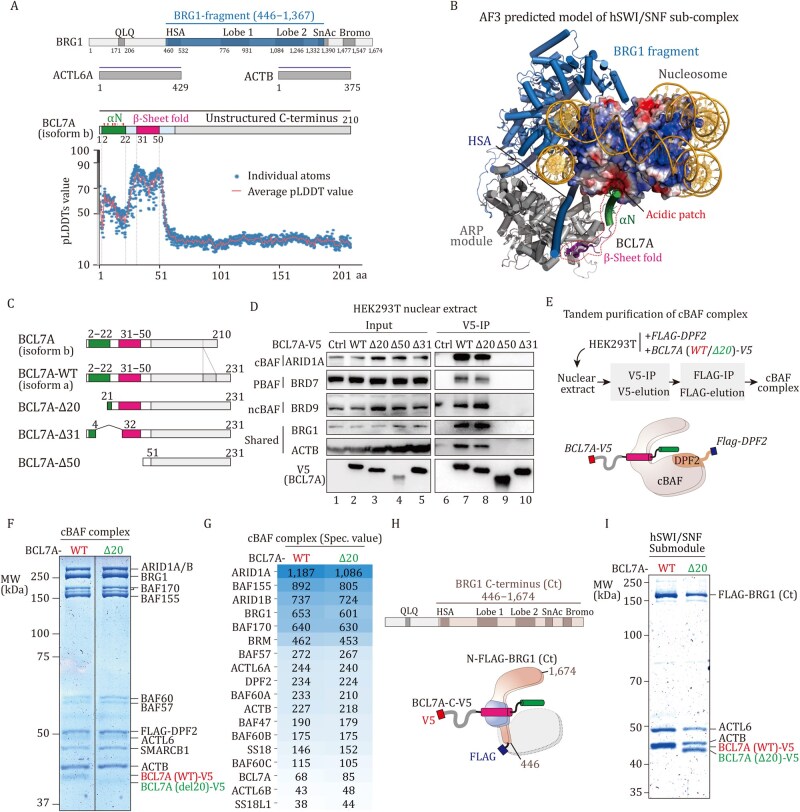
**BCL7A employs distinct structural domains for hSWI/SNF complex incorporation and nucleosome binding**. (A) Full-length ACTB, ACTL6A, BCL7A, and a fragment of BRG1 (top), along with a mono-nucleosome containing Wisdom’s 601 sequence and Xenopus core histones, were used to predict the structure of the nucleosome-bound human SWI/SNF subcomplex. Arginine and lysine residues in N-terminus of BCL7A are marked with red and pink arrows, respectively. The bottom panel shows predicted structural domains and pLDDT scores for BCL7A within the hSWI/SNF subcomplex. (B) Predictive model of the nucleosome-bound hSWI/SNF subcomplex, highlighting the N-terminus of BCL7A with a red dashed line. Histone colors indicate electrostatic potential, with red representing acidic regions. (C) Schematic representation of BCL7A (isoform a) WT and truncations used in this study. (D) Anti-V5 immunoprecipitation results showing interactions between V5-tagged BCL7A WT or truncations and hSWI/SNF complex subunits in HEK293T cells. (E) Workflow of V5-FLAG tandem purification (top) and a schematic of the BCL7A-containing cBAF complexes (bottom). (F) Coomassie blue staining of V5-FLAG tandem-purified cBAF complexes with BCL7A-WT or BCL7A-Δ20 on SDS-PAGE. Equal volume of each purified complex sample was resolved on the gel, and the concentration of cBAF complexes were quantified by comparing BRG1 band intensity to a BSA standard curve on the same gel. (G) Mass spectrometry analysis of purified cBAF complexes with BCL7A-WT or BCL7A-Δ20, using a color gradient to indicate spec. values, with darker gradients representing higher values. (H) Schematic of BRG1 C-terminus (top) and the tandem-purified hSWI/SNF submodule (bottom), with missing parts in gray and ACTB and ACTL6A/B in blue. (I) Coomassie blue staining of V5-tagged BCL7A and FLAG-tagged BRG1 C-terminus in tandem-purified hSWI/SNF submodule on SDS-PAGE.

BCL7A is a shared subunit of all three human SWI/SNF complex variants: cBAF, PBAF, and ncBAF. Previous studies using co-immunoprecipitation (co-IP), followed by Western blotting or mass spectrometry, showed that BCL7A interacts with the ATPase BRG1 (Mashtalir et al., 2018; Schick et al., 2019). However, detailed structural information of BCL7A in the context of hSWI/SNF complex remains elusive. To bridge this gap in knowledge, we utilized the AlphaFold3 algorithm (Abramson et al., 2024) to predict the structure of a core subcomplex, including a BRG1 fragment (amino acids 446–1,367, encompassing the HSA domain and helicase region), the ARP module (ACTL6A, ACTB, BCL7A), and a nucleosome core particle (NCP) consisted of Xenopus histones and Widom’s 601 DNA sequence (Fig. 1A). The high pLDDT score of the BCL7A N-terminal region indicates a robust and reliable structural prediction (Fig. 1A) and the structural model reveals a defined positively charged N-terminal α-helix (amino acids 2–22, αN) and a double-stranded β-sheet (amino acids 31–50, β-sheet fold) in this region (Fig. 1A and 1B). As expected, the unstructured C-terminal region of BCL7A is not visualized in this model (Fig. 1A). In addition, the model predicts that the BCL7A N-terminal α-helix, rich in arginine and lysine residues, binds stably to the acidic patch of H2A/H2B in the nucleosome while the β-sheet fold of BCL7A facilitates its interaction with ACTB and ACTL6A (Fig. 1B). Notably, the predicted structure of the BRG1 fragment, ACTB, ACTL6A, and NCP closely resembles the cryo-EM-determined structure (Fig. S1A) with the only exception of the BRG1 HSA domain. PAE analysis indicates high prediction confidence for the spatial arrangement of BCL7A’s N-terminal α-helix near the nucleosome and β-sheet near the ARP module (Fig. S1B–D). These critical structural regions are supported by consistently high pLDDT (predicted Local Distance Difference Test) scores (Fig. S1E–H), reflecting strong domain-level prediction ­confidence. Together, these metrics demonstrate the reliability of our predicted hSWI/SNF subcomplex model.

To systematically examine the role of the BCL7A N-terminal domain in its association with the hSWI/SNF complexes, we constructed several BCL7A truncations: deletion of the N-terminal α-helix (Δ20), a functional mutation associated with DLBCL (Δ31) (Baliñas-Gavira et al., 2020), and removal of the entire N-terminal domain (Δ50) (Fig. 1C). These BCL7A truncation proteins were tagged with a V5 epitope and overexpressed in HEK293T cells using lentiviral vectors. First, the interactions between BCL7A and key subunits ARID1A, BRD7, and BRD9 were confirmed using immunoprecipitation assays targeting the V5 tag, validating the BCL7A integration into the cBAF, PBAF, and ncBAF complexes, respectively (Fig. 1D). Notably, the deletion of the N-terminal α-helix (Δ20) did not affect the interaction between BCL7A and these complexes (Fig. 1D). In contrast, deletions of both the N-terminal β-sheet (Δ31) or the entire N-terminal domain (Δ50) disrupted the interaction between BCL7A and the hSWI/SNF complexes (Fig. 1D). These findings corroborate our structural prediction, indicating that while the N-terminal α-helix is not essential for the incorporation of BCL7A into hSWI/SNF, the β-sheet domain is required for these interactions.

To further examine the structural contribution of the BCL7A N-terminal domain, we employed a tandem purification strategy to study the integrity of the hSWI/SNF complexes. FLAG-tagged cBAF unique subunit DPF2 and V5-tagged wild-type (WT) or N-terminal truncated mutant (Δ20) of BCL7A were co-overexpressed in HEK293T cells via sequential lentiviral infections (Fig. 1E). The cBAF complexes containing either the WT or truncated BCL7A were then specifically isolated using V5-FLAG tandem affinity purification. The integrity and proper subunit stoichiometry of the purified complexes were assessed by Coomassie Blue staining, which showed no significant differences between the complexes containing WT and truncated BCL7A variants (Fig. 1F). Further mass spectrometry analysis confirmed that both WT and Δ20 BCL7A-containing cBAF complexes exhibited nearly identical subunit composition and relative abundance (Fig. 1G). These results are consistent with our immunoprecipitation results (Fig. 1D), indicating that the deletion of the N-terminal α-helix (Δ20) does not affect BCL7A’s ability to bind the hSWI/SNF complexes nor disturb their structural integrity.

In addition, our structural prediction indicates that BCL7A, in conjunction with ACTB and ACTL6A, specifically binds to the HSA domain of BRG1, forming a core subcomplex distinct from the previously reported structures of the cBAF, PBAF, or ncBAF complexes. To determine whether this partial complex—particularly with the N-terminal truncation of BRG1 that folds into the base module—can exist as a stable physical entity, we reconstituted this hSWI/SNF submodule. This submodule contains a FLAG-tagged BRG1 C-terminal construct (BRG1(Ct)), which includes only the HSA, helicase, SnAc, and bromodomain (Fig. 1H). BRG1(Ct) was overexpressed in HEK293T cells along with V5-tagged WT or N-terminal truncated BCL7A (Δ20), and the respective subcomplexes were isolated using V5-FLAG tandem affinity purification. The results demonstrate the formation of a stable subcomplex, with no significant compositional difference between the WT and Δ20 BCL7A variants (Fig. 1I). Together, our findings demonstrate that the N-terminal double-stranded β-sheet of BCL7A mediates its incorporation into hSWI/SNF by directly engaging the ACTB-ACTL6A dimer, which in turn associates with the HSA domain of BRG1. In contrast to the β-sheet, the α-helix of BCL7A shows dispensability for hSWI/SNF incorporation.

### The N-terminal α-helix of BCL7A plays a critical regulatory role in cBAF remodeling activity

The structural characteristics of the N-terminal α-helix (αN) of BCL7A includes the enrichment of five arginine residues (R4, R7, R11, R13, R20) and two lysine residues (K15, K19), which imparts a robust positive charge and is considered crucial for its interaction with the acidic patch of the nucleosome. This structural feature closely mirrors that of the C-terminal α-helix of SMARCB1 (Valencia et al., 2019), another integral component of the cBAF and PBAF complexes. Given that previous studies observed a higher prevalence of BCL7A in the cBAF compared with PBAF and ncBAF using glycerol gradient sedimentation and mass spectrometry (Mashtalir et al., 2018), we decided to focus on the cBAF complex for the functional study of BCL7A αN.

**Figure 2.**
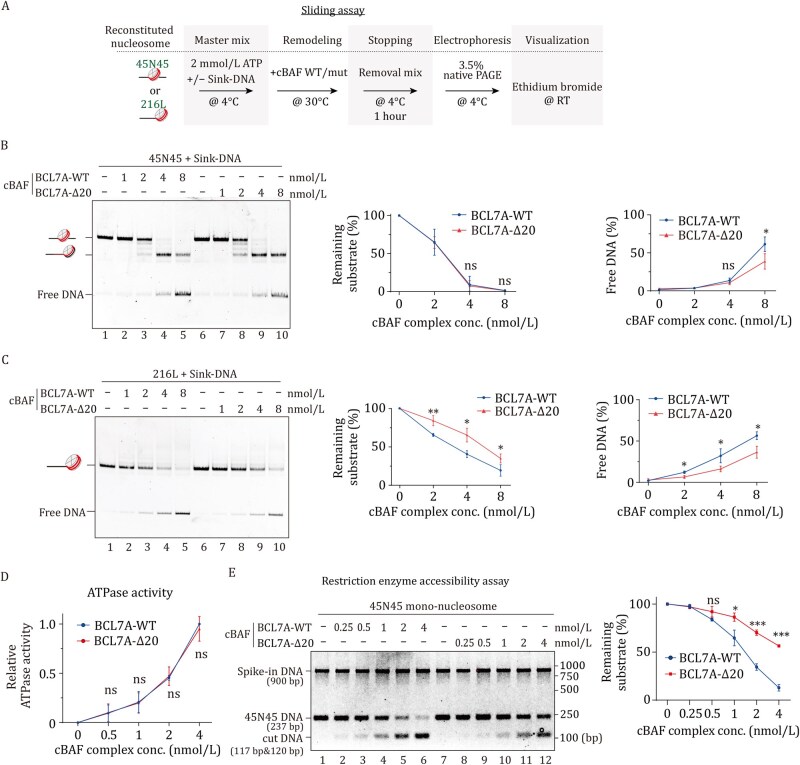
**Regulatory role of BCL7A’s N-terminal α-helix in cBAF remodeling activity**. (A) Workflow for the *in vitro* nucleosome sliding assay using either center-positioned 45N45 or end-positioned 216 L nucleosomes. (B) Representative image of the sliding assay with sink-DNA addition (left), and quantification of remaining substrate and free DNA product (right) using 20 nmol/L 45N45 nucleosome. (C) Representative image of the sliding assay with 20 nmol/L 216 L mono-nucleosome and sink-DNA (left), and quantification of the histone displacement ratio by measuring free DNA relative to input nucleosomes (right). (D) ATPase activity assay with cBAF complexes and 10 nmol/L 45N45 nucleosomes. Workflow shown in Fig. S2C. (E) Restriction enzyme accessibility assay with 10 nmol/L 45N45 nucleosomes and cBAF complexes (top), with quantification of remaining substrate normalized to spike-in DNA (bottom). Workflow shown in Fig. S2D. All data show mean ± SD from three individual replicates; *P* value calculated by unpaired Student’s *t* test. “ns”, not significant; *P < 0.05, **P < 0.01, ***P < 0.001.

To examine the chromatin remodeling activity of cBAF, we first reconstituted a center-positioned nucleosome (45N45) template *in vitro*, utilizing a 237-bp DNA sequence that incorporates the 147-bp Widom 601 sequence flanked by 45-bp linker DNA on each side, and HeLa core histones (Lee et al. 2013) ( Huh et al. 2012). The chromatin remodeling activity of the cBAF complex was evaluated by the sliding assay, which measures the histone octamer translocating toward the DNA ends and the histone displacement according to the electrophoretic mobility on native polyacrylamide gel (Fig. 2A). The result confirmed that the deletion of the BCL7A αN in the cBAF complex caused significant decrease in the consumption of end-positioned nucleosome, and decrease in the generation of free DNA compared with WT without affecting the depletion of center-positioned nucleosome substrate (Figs. 2B and S2A). To further elucidate the role of BCL7A αN in histone displacement, we reconstituted an end-positioned nucleosome template (216 L), consisting of the 147-bp 601 sequence with a 69-bp extension on only one side (Fig. 2C). This chromatin configuration provides an additional window into the dynamics of histone displacement. The results indicated that the absence of the BCL7A αN diminished the depletion of the 216 L substrate and reduced the production of free DNA (Fig. 2C). These findings collectively highlight the important function of the N-terminal α-helix of BCL7A in modulating histone displacement during cBAF chromatin remodeling.

We next tested whether the BCL7A αN modulated the motor activity of the BRG1 ATPase in the cBAF complex. For this purpose, we performed a quantitative ATPase assay using the 45N45 nucleosome template. However, the results showed no significant difference in ATPase activity (Figs. 2D and S2B), suggesting that αN does not directly influence the enzymatic function of BRG1 ATPase. We then speculated that BCL7A αN may regulate cBAF-mediated nucleosomal DNA accessibility. To this end, we conducted a restriction enzyme accessibility assay (REAA) and utilized a HhaI site proximal to the dyad axis of the 45N45 nucleosome. DNA accessibility of this site will allow the production of two distinct fragments of 120 and 117 bp, respectively. Interestingly, deletion of the BCL7A N-terminal α-helix markedly decreased the production of these fragments (Figs. 2E and S2C), underscoring the importance of BCL7A αN in facilitating cBAF-mediated DNA accessibility at the nucleosome dyad. Notably, the REAA measures combined effects of both nucleosome sliding and complete dissociation. The impaired REAA efficiency caused by αN deletion (Fig. 2E) correlates well with the reduced activity observed in sliding assays (Fig. 2B), suggesting αN primarily facilitates the remodeling outcomes rather than the initial ATPase-driven mobilization.

### BCL7A works together with SMARCB1 in modulating hSWI/SNF activity

**Figure 3.**
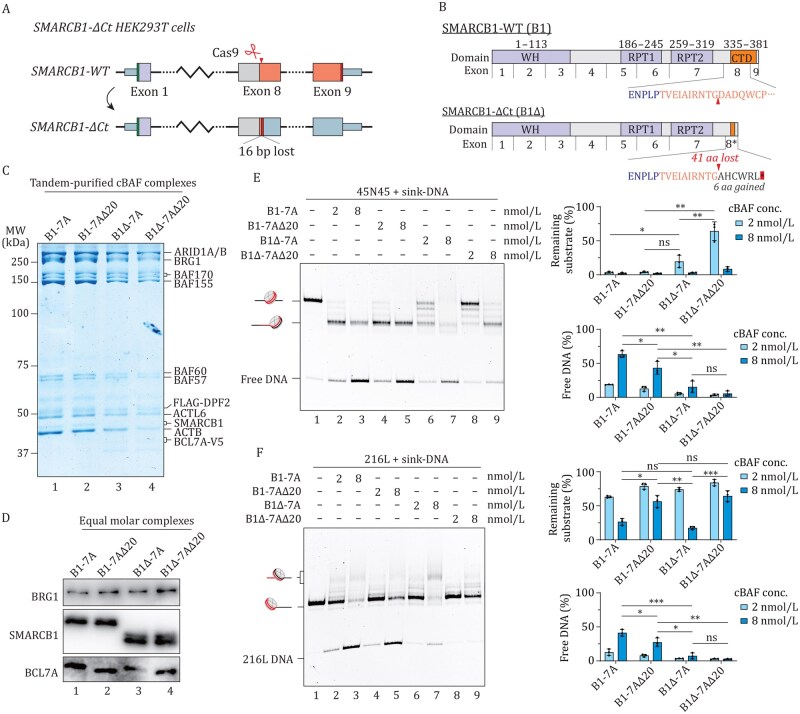
**SMARCB1 and BCL7A work together in modulating hSWI/SNF chromatin remodeling activity**. (A) Generation of a HEK293T cell line with a C-terminal truncation (ΔCt) in SMARCB1 using CRISPR-Cas9, targeting exon 8 and resulting in a 16-bp deletion. (B) Schematic of SMARCB1 protein showing domains and exon organization in WT and C-terminal mutant (B1Δ), highlighting the loss of the CTD due to a 16-nt deletion causing a frameshift and premature translation termination. (C) Coomassie-stained SDS-PAGE of V5-BCL7A and FLAG-DPF2 in cBAF complexes purified from WT and SMARCB1-ΔCt HEK293T cells, with cBAF complex concentration quantified by ARID1A band intensity. (D) Western blot analysis of purified cBAF complexes at equal molarity. (E) Representative image of an *in vitro* sliding assay with indicated cBAF complexes and 20 nmol/L 45N45 nucleosome (left), with normalized intensities of remaining substrate and free DNA product shown as sliding and histone displacement ratios, respectively (right). (F) Representative image of an *in vitro* sliding assay with indicated cBAF complexes and 216 L nucleo­some (left), with quantification of remaining substrate (right). All data show mean ± SD from three individual replicates; P value calculated by unpaired Student’s *t* test. “ns”, not significant; *P < 0.05, **P < 0.01, ***P < 0.001.

The structural models of cBAF and PBAF complexes highlight the role of the CTD of SMARCB1 in binding to the nucleosome acidic patch (He et al., 2020; Valencia et al., 2019; Yuan et al., 2022), thereby stabilizing the complex’s interaction with chromatin. Our structural prediction indicates that BCL7A may interact with the acidic patch on the same side of the nucleosome facing SMARCB1 (Fig. S1A), suggesting a potential competitive or cooperative relationship between these two functional modules within the same complex. To explore the roles of these arginine anchors in chromatin remodeling, we engineered cBAF complexes with various combinations of arginine anchor mutations in SMARCB1 and/or BCL7A. To this end, we first utilized CRISPR-Cas9 editing to create a deletion in the SMARCB1 CTD in HEK293T cells (Fig. 3A). Following transfection with Cas9 and guide RNAs targeting the eighth exon of SMARCB1, single-cell cloning and expansion were performed. Western blotting confirmed the presence of a truncated SMARCB1 variant (Fig. S3A), while sequencing of genomic DNA from this clone revealed a 16-base pair deletion in the eighth exon (Figs. 3A and S3B), which leads to a frameshift mutation and premature termination of translation (Figs. 3B and S3C). We then overexpressed FLAG-tagged DPF2, a unique subunit of cBAF, and V5-tagged BCL7A in the background of either WT or SMARCB1 CTD-deleted HEK293T cells to purify different forms of cBAF complexes (Fig. S3D and S3E). Despite a slight decrease of yield of complexes with SMARCB1 or BCL7A deletions, the subunit composition of cBAF remained consistent (Fig. 3C). Western blot analyses provided validation for the presence of the SMARCB1 and BCL7A variants in these purified complexes (Fig. 3D). Molar concentration of each form of the complexes was determined by quantification of the ARID1A on Coomassie blue-stained SDS-PAGE and the same molar of the complexes was used in following assays.

We next assessed the impact of these mutation combinations on cBAF chromatin remodeling activity using the 45N45 nucleosomal template. Similar to the results in Fig. 2, the removal of BCL7A αN selectively inhibited histone displacement without affecting the basic sliding activity of cBAF (Fig. 3E, compare lanes 3 and 5). SMARCB1 CTD can also contribute to cBAF’s remodeling efficiency though through different mechanisms indicated by the increased presence of residual substrate under identical complex concentration (Fig. 3E, compare lanes 3 and 7). cBAF devoid of both BCL7A and SMARCB1 arginine anchors showed a substantial decrease in remodeling efficiency, as shown by diminished substrate depletion, reduced number of fully-slid nucleosomes, and lower level of free DNA (Fig. 3E, compare lanes 3 and 9). Importantly, the B1Δ-7AΔ20 double mutant complex displayed even weaker sliding activity than the B1-7AΔ20 cBAF complex (Fig. 3E, compare lanes 5 and 9), suggesting that SMARCB1 has BCL7A αN-­independent functions in supporting nucleosome sliding. Moreover, the deletion of the SMARCB1 CTD also impaired histone displacement on end-positioned nucleosome substrates (Figs. 3F and S4A). In this scenario, the progression of nucleosome sliding events proceeds through intermediate phases, as evidenced by the presence of a slowly migrating, centrally localized nucleosome population (Fig. 3F, lane 7, and Fig. S4A, lanes 4 and 5), before ultimately resulting in histone displacement. This is consistent with previous observations (Gutierrez et al., 2007). Compared with B1-7AΔ20 cBAF, B1Δ-7A appears to show much lower sliding and histone displacement activity, with more nucleosomes being trapped at the intermediate centrally positioned stages (Fig. 3F, lane 5 vs. lane 7). The double mutant B1Δ-7AΔ20 complex showed the most reduced remodeling activity (Fig. 3F, lane 9). These results suggest that SMARCB1 and BCL7A work together to regulate cBAF chromatin remodeling activity.

### The arginine anchor of BCL7A is essential for tumor suppression

BCL7A has been identified as a tumor suppressor in DLBCL (Baliñas-Gavira et al., 2020), which is the most prevalent subtype of lymphoid malignancy, and ∼30% of its patients fail to respond to current therapies (Kober-Hasslacher and Schmidt-Supprian, 2019; Li et al., 2018; Lohr et al., 2012). Recent whole-genome sequencing has uncovered a high frequency of mutations in genes associated with epigenetic regulation in DLBCL (Pasqualucci and Dalla-Favera, 2018; Reddy et al., 2017; Schmitz et al., 2018; Steen et al., 2021). Notably, over 50% of germinal center B-cell-like (GCB)-DLBCL cases show mutation of subunits of the human SWI/SNF chromatin remodeling complex, including BRG1 (SMARCA4), ARID1A, and BCL7A (Baliñas-Gavira et al., 2020; Kadoch et al., 2013). For example, a DLBCL mutation affecting BCL7A intron splicing produces a truncated protein variant that lacks N-terminal 5–31 amino acid residues, which is unable to incorporate into the hSWI/SNF complex. The re-expression of WT BCL7A in tumor cells harboring this mutation significantly inhibits tumor growth both *in vitro* and *in vivo*, underscoring the critical role of BCL7A in tumor suppression (Baliñas-Gavira et al., 2020). Given that this BCL7A DLBCL mutation also occurs in the similar region of the BCL7A αN that our work predicted to interact with the nucleosome acidic patch, we speculated that the BCL7A αN may be crucial for its tumor-suppressive function in DLBCL.

To investigate the regulatory mechanisms of BCL7A in DLBCL, we chose to focus on the DLBCL cell line OCI-LY1 which harbors a previously identified heterozygous BCL7A mutation: one allele with a start codon mutation disrupting translation, and the other with a 5′-splicing site mutation in the first intron, leading to a truncated protein missing amino acids 5–31 due to cryptic splicing (Fig. S5A) (Baliñas-Gavira et al., 2020). These mutations were verified using reverse transcription polymerase chain reaction (RT-PCR) and DNA sequencing. The results revealed two distinct lengths: a WT transcript and a shorter isoform, which differs from the single transcript observed in other DLBCL cell lines such as OCI-LY10, DB, and Su-DHL-4 (Fig. S5B). Both transcripts contained a mutation in the start codon of the longer transcript and an 81-nt deletion in the shorter transcript, corresponding to the loss of amino acids 5–31 (Fig. S5C). Western blot analysis showed that OCI-LY1 cells exhibited lower total BCL7A protein and an isoform with reduced molecular weight, consistent with the predicted truncation (Fig. S5D). These findings confirm the loss-of-function BCL7A mutations in the OCI-LY1 cell line, validating its use for further functional studies.

**Figure 4.**
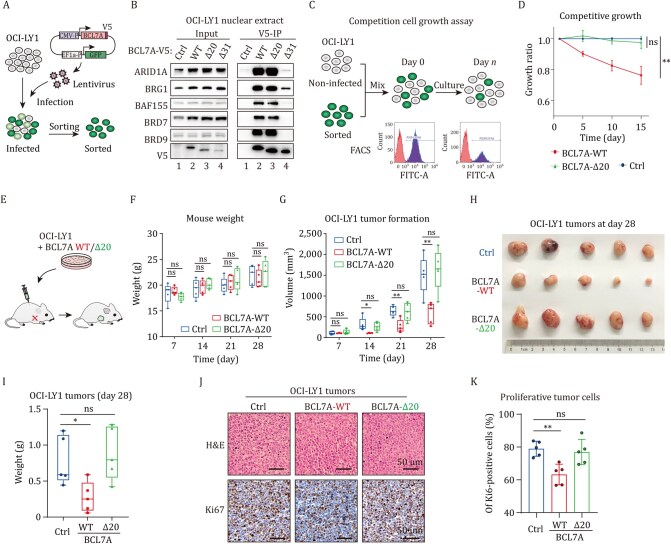
**The N-term α-helix of BCL7A is essential for DLBCL tumor suppression**. (A) Diagram of BCL7A-V5 lentiviral expression constructs with GFP selection in OCI-LY1 cells. (B) Anti-V5 immunoprecipitation demonstrating interactions between V5-tagged BCL7A (WT or truncated) and hSWI/SNF complex subunits in OCI-LY1 cells. (C) Diagram of the competition cell growth assay in OCI-LY1 cells. (D) Competition cell growth assay results showing compromised cell growth suppression by N-terminal α-helix truncation of BCL7A in OCI-LY1 cells. Data from three biological replicates are presented as mean ± SD. Differences on day 15 were analyzed using an unpaired Student’s *t* test. (E) Diagram of the subcutaneous tumor formation assay in nude mice. (F) Boxplots of mouse body weight during the *in vivo* tumor formation, presented as median ± IQR; *n* = 5. (G) Tumor size measurements during the assay. (H) Images of dissected tumors at day 28 of the assay. (I) Tumor weight measurements at experimental endpoint (day 28). (J) Representative images of H&E and Ki67 immunostaining in OCI-LY1 tumors under Ctrl, BCL7A-WT, or BCL7A-Δ20 conditions. (K) Quantification of Ki67-positive cells in OCI-LY1 tumors, with data from five random visual fields per group, analyzed using a double-blind method and presented as mean ± SD. Statistical significance was determined by unpaired Student’s *t* test. “ns”, not significant; *P < 0.05, **P < 0.01, ***P < 0.001.

V5-epitope-tagged BCL7A and its truncated variants were stably overexpressed in OCI-LY1 cells using an established lentiviral infection protocol (Shen et al., 2022). The expression vector also contains green fluorescent protein (GFP) which facilitates cell sorting via flow cytometry (Fig. 4A). We first assessed the interaction between BCL7A and other subunits of hSWI/SNF complex by co-immunoprecipitation assays using the anti-V5 antibody (Fig. 4B). We found that WT BCL7A interacted with BRG1, ARID1A, BRD7, and BRD9, indicating an association with the cBAF, PBAF, and ncBAF complexes. In contrast, the BCL7A-Δ31 protein that is lacking amino acids 5–31 exhibited no binding to these subunits except weak interaction with BRG1, suggesting that disruption of the BCL7A N-terminal region abolished its interaction with the hSWI/SNF complex (Fig. 4B). However, BCL7A-Δ20, missing only the N-terminal α-helix (amino acid residues 1–20), maintained WT level of interactions with these subunits. V5-epitope signal in the IP inputs confirmed comparable expression levels of both BCL7A WT and Δ20 proteins. These results suggested that the N-terminal α-­helix is not critical for association with hSWI/SNF in OCI-LY1 cells, consistent with the result in HEK293T (Fig. 1D).

We next evaluated the role of BCL7A αN on tumor cell proliferation using competition cell growth assays (Fig. 4C). In this assay, OCI-LY1 cells co-expressing GFP and V5-tagged WT BCL7A or N-terminal truncated BCL7A-Δ20 were mixed with an equal number of non-GFP-expressing OCI-LY1 cells as an internal control. The influence of BCL7A variants on cell growth was measured by the relative proportion of GFP-positive cells using flow cytometry (Fig. 4C). Indeed, we found that expression of WT BCL7A led to a significant suppression of OCI-LY1 cell proliferation, whereas the cells expressing the truncated BCL7A-Δ20 variant showed no such inhibitory effects (Fig. 4D). This result substantiates the critical role of BCL7A αN in tumor suppression of DLBCL.

We next tested the tumor-suppressive role of BCL7A αN *in vivo* using subcutaneous tumor formation assays on nude mice (Fig. 4E). Throughout the experiment, the body weight of the mice showed no significant intergroup differences which ensured the reliability of the tumor measurements (Fig. 4F). We found that WT BCL7A (BCL7A-WT) markedly decreased the OCI-LY1 tumor volume while deletion of BCL7A αN abolished this tumor suppressor activity (Fig. 4G). Consistently, the isolated tumor at 28-day post-inoculation showed that BCL7A-WT expression significantly reduced the tumor size compared with BCL7A-Δ20 or empty vector (Fig. 4H and 4I). Subsequent histological staining of the excised tumors displayed characteristic DLBCL features, including large nuclei, eosinophilic nucleoli, vacuolated chromatin, and relatively abundant cytoplasm (Figs. 4J and S5E), consistent with the established morphological criteria for DLBCL (Li et al., 2018). In addition, immunohistochemical analysis of Ki67, a proliferation marker, revealed that the BCL7A-WT expressing tumor cells showed significantly lower ratios of Ki67-positive cells (Figs. 4J, 4K and S5F). In contrast, the Ki67 positivity in the BCL7A-Δ20 group did not differ significantly from the control (Fig. 4K). These findings together support the indispensable role of BCL7A αN in tumor-suppressive activity for DLBCL.

### The arginine anchor of BCL7A mediates target gene transcription in DLBCL

**Figure 5.**
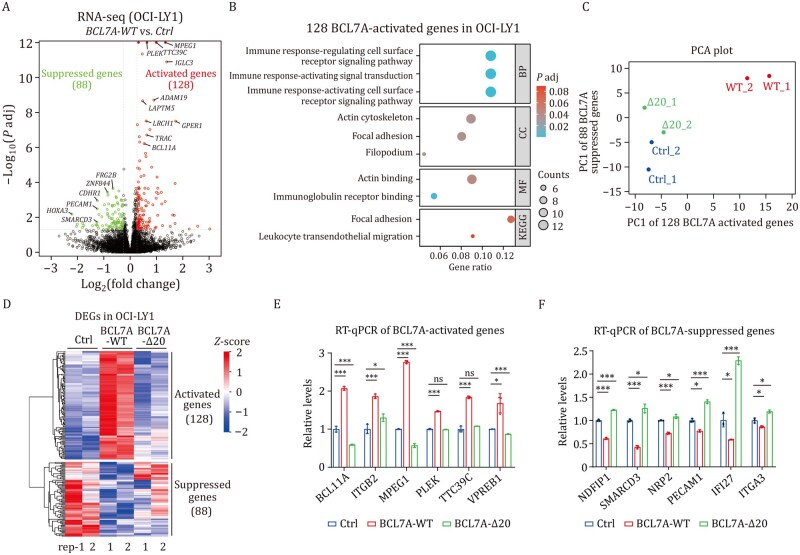
**Role of BCL7A’s N-terminal α-helix in target gene transcription in DLBCL**. (A) Volcano plot of RNA-seq data comparing gene expression between BCL7A-WT expressed and control OCI-LY1 cell lines, with upregulated genes in red and downregulated genes in green. (B) Top enriched Gene Ontology (GO) terms for the 128 genes activated by BCL7A-WT. (C) PCA plot illustrating the separation of control, BCL7A-WT, and Δ20 samples based on PC1 for the expression of 128 activated and 88 suppressed genes. (D) Heatmap displaying gene expression differences between control, BCL7A-WT and Δ20 in OCI-LY1 cells. (E and F) RT-qPCR validation of selected BCL7A-­activated (E) and suppressed genes (F), with statistical significance determined by unpaired Student’s *t* test (*n *= 3); “ns”, not significant; *P < 0.05, **P < 0.005, ***P < 0.001.

Given the tumor suppressor effect of BCL7A on DLBCL, we next employed RNA-seq to study its regulation on DLBCL target genes. To this end, RNA was isolated from OCI-LY1 cells expressing either WT or BCL7A-Δ20 (Fig. 4A and 4B). Differential expression analysis, as shown in a volcano plot, indicated that WT BCL7A significantly upregulated 128 genes, including *MPEG1* and *PLEK*, and downregulated 88 genes, including *HOXA3* and *SMARCD3* (Fig. 5A). Subsequent GO analysis of the upregulated genes highlighted BCL7A’s involvement in immune response–related processes, particularly in regulating cell surface receptor signaling pathways involved in immune responses (Fig. 5B). In addition, KEGG pathway analysis linked BCL7A activity to focal adhesion and leukocyte transendothelial migration, suggesting its role in immune cell trafficking and adhesion. Interestingly, PCA analysis showed similar expression profiles of the BCL7A-Δ20 mutant and the control, indicating that BCL7A αN is indispensable for its function (Fig. 5C). A heatmap of differentially expressed genes revealed two distinct clusters: one cluster contained 128 BCL7A-activated genes dependent of BCL7A αN, while the other cluster included 88 BCL7A-repressed genes dependent or independent of the BCL7A αN (Fig. 5D). However, OCI-LY1 cells expressing the N-terminal deletion mutant (BCL7A-Δ20) showed fundamentally different transcriptional outcomes, with 90 significantly activated and 92 suppressed genes compared with controls (Fig. S5G and S5H). Notably, less than 10% of DEGs overlapped between WT and Δ20 conditions (Fig. S5I). This divergence was further underscored by GO term analysis, where top enriched pathways in Δ20-regulated genes (response to virus and carbohydrate binding) showed minimal similarity to WT-associated terms (Fig. S5J). Collectively, these data demonstrate that the BCL7A αN plays a more important role in transcriptional activation.

To validate these findings, RT-qPCR was conducted on selected genes from the two clusters, including *BCL11A*, *ITGB2*, *MPEG1*, *PLEK* (activated genes), and *NDFIP1*, *SMARCD3*, *NPR2*, *PECAM1* (repressed genes). The RT-qPCR results corroborated the RNA-seq data, confirming the expression trends observed across different experimental groups (Fig. 5E and 5F). These results collectively underscore the pivotal role of BCL7A, particularly αN, in modulating gene expression, thereby potentially contributing to immune regulation and tumor suppression in DLBCL. While our pathway analysis identified biologically relevant signatures among the differently expressed genes, we acknowledge that comprehensive mapping of the downstream signaling networks mediating growth inhibition would require substantial future investigation beyond the scope of our current mechanistic study on chromatin remodeling. Nevertheless, these findings exhibit strong consistency with the results from cBAF complex remodeling activity assays *in vitro* and tumor phenotype studies *in vivo*, further supporting the functional significance of the BCL7A arginine anchor in DLBCL tumor suppression.

### Arginine anchor deficiency reduces BCL7A genome-wide occupancy in DLBCL

**Figure 6.**
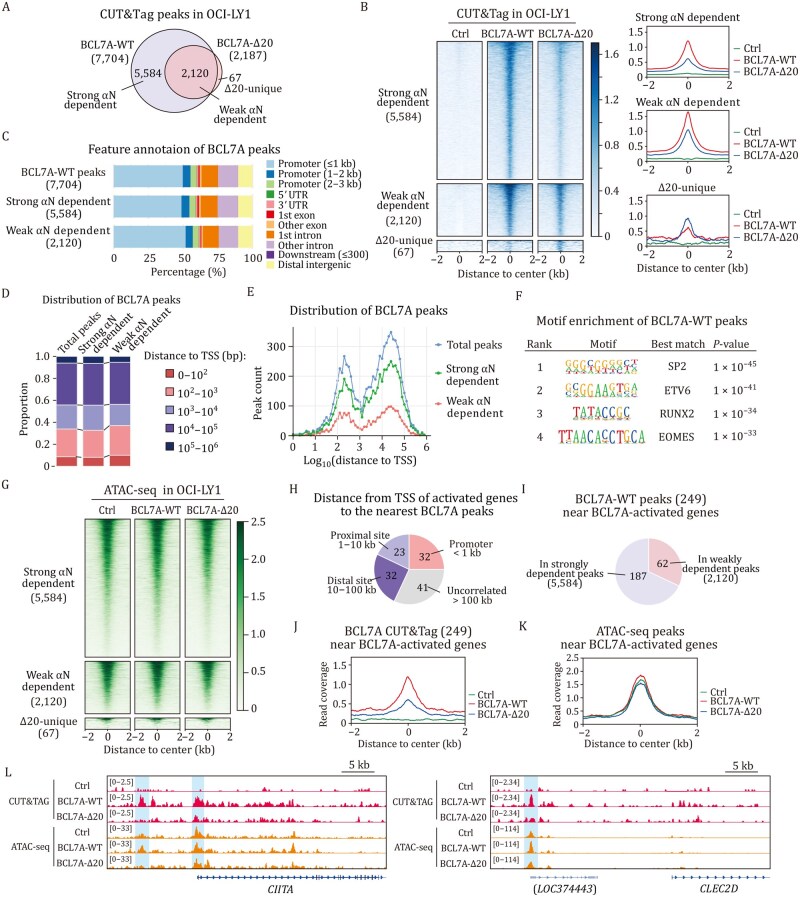
**The genomic localization of BCL7A and its functional implications in chromatin remodeling within OCI-LY1 cells**. (A) Venn diagram showing overlap between BCL7A-WT and Δ20 CUT&Tag peaks. The significant peaks in each group were determined against control (*P < *0.05). The peaks were categorized by dependence on the N-terminal α-helix for chromatin occupancy, with significance. (B) Heatmap (right) and binding profiles (left) depicting occupancy of BCL7A-WT and BCL7A-Δ20 at sites with varying dependency on the N-terminal α-helix. (C) Feature annotation of BCL7A CUT&Tag peaks for different groups. (D) Distribution of distances from BCL7A binding peaks to the nearest TSS. (E) Frequency distribution of distances from BCL7A binding peaks to the nearest TSS. (F) Top enriched transcription factors from motif analysis of BCL7A-WT binding peaks. (G) Heatmaps of ATAC-seq results showing chromatin accessibility at BCL7A-associated peaks in OCI-LY1. (H) Distance distribution from TSS of BCL7A-activated genes to the nearest BCL7A peak, involving 249 peaks near 87 of the 128 activated genes. (I) Venn diagram classifying 249 BCL7A-WT binding peaks near BCL7A-activated genes. (J) Summary metaplots of BCL7A occupancy near activated genes under Ctrl, BCL7A-WT, and BCL7A-Δ20 conditions. (K) Summary metaplots showing genomic accessibility at 249 BCL7A-WT binding peaks across different conditions. (L) Representative CUT&Tag and ATAC peaks for BCL7A WT and Δ20 near activated genes in OCI-LY1.

To test how αN-directed BCL7A-nucleosome interaction may influence BCL7A chromosome association *in vivo*, we employed CUT&Tag with a V5-epitope tag to map BCL7A binding sites across the genome in OCI-LY1 cells. We detected 7,704 WT BCL7A (BCL7A-WT) binding peaks, designated as total peaks (Fig. 6A). In contrast, the BCL7A-Δ20 mutant exhibited a significant reduction in peak number (Fig. 6A) with only 2,120 peaks common with BCL7A-WT (termed weak αN dependent peaks), while the missing 5,584 peaks were termed strong αN dependent peaks. The BCL7A-Δ20 mutant also had 67 unique peaks which were termed Δ20-unique peaks (Fig. 6A). More detailed analysis revealed that, although not formally identified in the peak calling for the BCL7A-Δ20 mutant, the 5,584 WT-specific peaks still exhibited residual binding by BCL7A-Δ20, albeit at reduced levels (Fig. 6B). Additionally, the BCL7A-Δ20 showed a modest decrease in peak intensity within the 2,120 overlapping peaks. These findings suggest that instead of recruiting the hSWI/SNF complex to specific genomic locations, BCL7A αN may enhance and stabilize the binding of the hSWI/SNF to these loci.

To further delineate the genomic distribution of BCL7A binding sites, we analyzed the detailed location characteristics of these peaks across the genome. Annotation of genomic features revealed that a substantial proportion of BCL7A peaks were localized to proximal promoters (48.8%), followed by introns (26.7%) and distal intergenic regions (10.4%) (Fig. 6C). This distribution pattern was consistent between WT and BCL7A-Δ20 specific peaks, once more indicating a similar genomic localization independent of αN. Moreover, we assessed the proximity of BCL7A peaks to transcription start sites (TSS) of adjacent genes and found that the distribution of peaks relative to TSS distances was uniform in WT and mutant BCL7A, with a significant portion of peaks within close proximity to TSS (Fig. 6D and 6E). These findings suggest that BCL7A-containing hSWI/SNF preferentially targets regulatory regions near gene promoters, a pattern that holds true across different BCL7A variants. Motif analysis identified the enrichment of binding sites of several transcription factors in this region, including SP2, ETV6, RUNX2, and EOMES (Fig. 6F), further supporting their potential collaboration with BCL7A in modulating gene transcription in DLBCL.

To investigate the interplay between BCL7A occupancy and hSWI/SNF-mediated chromatin accessibility, we conducted ATAC-seq on OCI-LY1 cells transfected with either WT BCL7A (BCL7A-WT) or BCL7A-Δ20 (Δ20). Our analysis focused on chromatin accessibility in regions characterized by differential BCL7A binding. Results indicated a modest increase in chromatin accessibility in cells expressing BCL7A-WT, whereas cells expressing BCL7A-Δ20 showed a slight reduction in accessibility (Figs. 6G and S6A). These observations implied that BCL7A αN may subtly modulate chromatin accessibility, once more suggesting a fine-tuning regulatory rather than hSWI/SNF recruitment. Additionally, we noted that genomic regions harboring weak αN-dependent BCL7A peaks exhibited significantly enhanced chromatin accessibility compared with those with strong αN-dependent peaks. This pattern suggests that αN of BCL7A may be more important in areas of inherently lower chromatin openness.

Our transcriptomic analyses suggested that the αN terminal domain of BCL7A is crucial for the upregulation of specific genes. We further investigated this by examining the proximity of TSS of these activated genes to WT BCL7A-binding sites. Our findings indicate that among the upregulated genes, 32 were located within less than 1 kb, 23 within 1–10 kb, and 32 within 10–100 kb from a BCL7A peak, aligning with the general genomic distribution of BCL7A-binding sites (Fig. 6H). We analyzed 249 BCL7A peaks near the activated genes and identified that 75.1% (187 peaks) exhibited strong αN dependence, whereas 24.9% (62 peaks) showed weak αN dependence (Fig. 6I). Significantly, the occupancy of BCL7A-WT at these sites was considerably higher than that observed in controls and the BCL7A-Δ20 mutant (Figs. 6J, 6L and S6B). In cells expressing BCL7A-Δ20, chromatin accessibility was slightly reduced compared with those expressing BCL7A-WT (Figs. 6K, 6I and S6C), emphasizing the critical role of BCL7A binding in gene transcription and the critical function of the αN domain in this process. However, the disparity between BCL7A chromatin occupancy and transcriptional activation suggests a nuanced, context-dependent regulation. This implies that while BCL7A-mediated nucleosome remodeling may determine local chromatin architecture and accessibility, the final transcriptional outputs and the tumor-suppressive impacts of BCL7A are influenced by the interplay with other transcriptional regulators, the availability of cofactors, and downstream signaling pathways. This complex regulatory framework potentially accounts for the inconsistent expression of BCL7A’s tumor suppressor activity across different cellular environments.

### Recurrent mutations reveal the essential role of BCL7A’s arginine anchor in DLBCL tumor suppression

**Figure 7.**
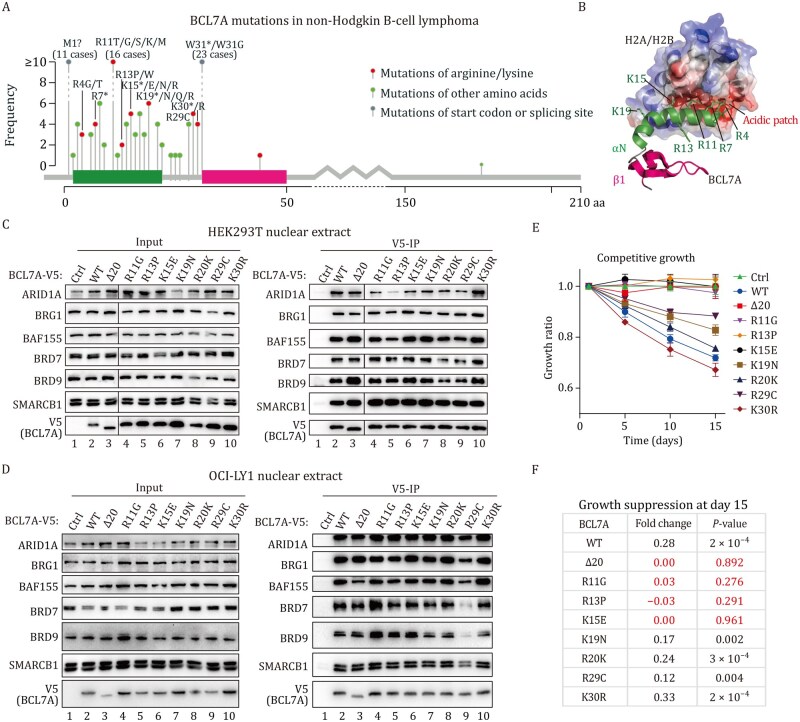
**Recurrent mutations reveal the essential role of BCL7A’s arginine anchor in DLBCL tumor suppression**. (A) Overview of BCL7A point mutations identified across 2055 non-Hodgkin lymphoma cases, including 1,295 DLBCL and 760 mature B-cell malignancies. (B) AlphaFold-predicted interaction between BCL7A’s N-terminal domain and the acidic patch of H2A/H2B within the hSWI/SNF sub-complex, highlighting arginine and lysine residues in green sticks (related to Fig. 1B). (C) Anti-V5 immunoprecipitation demonstrating that V5-tagged BCL7A mutations do not affect interactions with hSWI/SNF complexes in HEK293T cells. (D) Anti-V5 immunoprecipitation assessing the impact of BCL7A mutations on its interaction with hSWI/SNF complexes in OCI-LY1 cells. (E) Competitive cell growth assay showing the effect of N-terminal BCL7A mutations on tumor-suppression in OCI-LY1 cells, with data shown as mean ± SD from three independent experiments. (F) Analysis of fold change in competitive cell growth relative to control, with statistical significance determined by unpaired Student’s *t* tests. Mutations R11G, R13P, and K15E, which impair tumor-suppressive activity, are marked in red (no significant difference vs. control).

Our results highlight the critical role of BCL7A αN in regulating gene transcription and tumor suppression. To assess its clinical relevance, we analyzed mutations associated with BCL7A in 2,055 non-Hodgkin B-cell lymphoma cases, including 1,295 cases of DLBCL and 760 cases of mature B-cell malignancies, using the cBioPortal database(Cerami et al., 2012) (Fig. 7A). We identified 131 cases with mutations impacting the BCL7A protein sequence, predominantly within the first exon that encodes the N-terminal 30 amino acids. Notably, mutations in arginine or lysine residues were observed in 45 cases, highlighting their significance in DLBCL pathogenesis. The clinic data correlated with our structural predictions indicated that the BCL7A αN functions as an arginine anchor, facilitating interactions with the acidic patch of the nucleosome (Figs. 1B and 7B) and modulating hSWI/SNF activity.

To explore the functional implications of these mutations, we focused on 7 arginine and lysine residues (R11, R13, K15, K19, R20, R29, and K30), which are recurrently mutated in DLBCL. We introduced point mutations at these locations in BCL7A and expressed the variants in HEK293T (Fig. 7C) and OCI-LY1 cells (Fig. 7D). Immunoprecipitation assays indicated that these mutations generally did not disrupt the interaction of BCL7A with hSWI/SNF complexes, with the exception of R29C, which partially reduced BCL7A binding with multiple subunits, likely due to its impact on BCL7A β-sheet formation (Fig. 7D). Competitive growth assays of OCI-LY1 cells showed that mutations at R11, R13, and K15 abolished BCL7A’s ability to suppress cell proliferation, whereas mutations at K19, R20, R29, and K30 retained partial growth-inhibitory activity (Fig. 7E and 7F). Notably, structural predictions using AlphaFold suggested that R11 serves as an arginine anchor, interacting with the nucleosome acidic patch, while other residues stabilize the α-helix structure and promote nucleosome binding through their positive charge (Fig. 7B). Indeed, naturally occurring DLBCL-associated mutations (R13P and K15E) disrupted the α-helical conformation (Fig. S7A and S7B), indicating that the tumor-suppressive function of the BCL7A arginine anchor was contingent upon maintaining its secondary α-helical structure. The consistency between structural predictions and functional assays strongly implicates nucleosome anchoring in BCL7A’s tumor-suppressive activity.

DLBCL exhibits a specific dependency on BCL7A, but not its paralogs BCL7B and BCL7C, prompting our investigation into the unique functions of BCL7A across a broad disease spectrum. Alin analysis of 1,673 tumor cell lines, categorized by lineage, reveals that BCL7A expression is significantly elevated in lymphoid (186 cases) and peripheral nervous system (45 cases) lineages compared with BCL7B and BCL7C, which display relatively uniform expression across all lineages (Fig. S8A and S8B). Additionally, within various immune cell types, BCL7A is predominantly expressed in B cells, especially naïve B cells, in contrast to the more evenly distributed expression of BCL7B and BCL7C (Fig. S8C). This lineage-specific expression pattern supports the hypothesis that BCL7A has evolved specialized chromatin remodeling function, which is especially critical in the context of lymphoid malignancies. In contrast, BCL7B and BCL7C appear to fulfill more generalized roles. These observations not only highlight the unique importance of BCL7A in lymphoid malignancies but also bolster its potential as a therapeutic target in DLBCL.

## Discussion

The hSWI/SNF chromatin remodeling complexes regulate chromatin accessibility and gene expression through evolutionarily conserved mechanism (Clapier et al., 2017), which includes recognition of modified histone tails via bromodomains or chromodomains (Mashtalir et al., 2021), helicase-mediated interactions with nucleosomal DNA (Han et al., 2020; Mashtalir et al., 2020; Wang et al., 2022; Ye et al., 2019; Yuan et al., 2022), and binding to the nucleosome acidic patch through the Snf2 ATP coupling (SnAc) domain or post-SnAc regions of BRG1/PBRM (Han et al., 2020; He et al., 2020; Mashtalir et al., 2020; Sen et al., 2013; Wang et al., 2022; Yuan et al., 2022; Ye et al., 2019). Additionally, the non-catalytic subunit SMARCB1 employs an arginine anchor to bind the acidic patch, further stabilizing the complex on chromatin (He et al., 2020; Mashtalir et al., 2020; Wang et al., 2022; Yuan et al., 2022). Although the direct binding of Snf5 (the yeast homolog of SMARCB1) to the nucleosome acidic patch has not been explicitly observed in the latest cryo-EM structures of yeast SWI/SNF and RSC complexes, Snf5 is spatially proximal to the acidic patch and plays a critical role in the activity and function of these chromatin remodeling complexes (Dechassa et al., 2008; Han et al., 2020; Sen et al., 2017; Ye et al., 2019). Arginine anchor-acid patch interaction is especially significant in human malignancies, where dysregulation of SMARCB1 has profound consequences. For instance, SMARCB1 loss drives the development of malignant rhabdoid tumors (RT) (Kim and Roberts, 2014; Nakayama et al., 2017; Wang et al., 2017), and its CTD mutations impair neural differentiation(Valencia et al., 2019). Similarly, in synovial sarcoma, the SS18-SSX fusion protein competitively binds the acidic patch, displacing SMARCB1 and disrupting chromatin remodeling (Kadoch and Crabtree, 2013; Li et al., 2021; McBride et al., 2018).

Our study reveals a previously unrecognized arginine anchor in BCL7A, a non-catalytic component of human SWI/SNF complexes, which engages with the nucleosome acidic patch. We employ structural predictions along with biochemical and functional assays to establish the pivotal role of the BCL7A arginine anchor in chromatin remodeling and its tumor-suppressive properties. Our data indicate that this anchor facilitates BCL7A’s attachment to the nucleosome, influences histone displacement, and modulates transcriptional activity *in vivo*. The disruption of this anchor, by either truncation or by introducing mutations commonly found in DLBCL such as R11G, R13P, K15E, leads to a loss of BCL7A’s tumor-suppressive function while not impeding its integration into the hSWI/SNF complex. These observations underscore BCL7A’s critical role in modulating chromatin architecture and illuminate the functions of non-catalytic subunits within the human SWI/SNF complexes. Although our results confirm the tumor-suppressive effect of WT BCL7A in functional deficiency models, it is important to acknowledge the extensive genetic diversity in DLBCL. The specific phenotypes observed are indicative of BCL7A’s function in restoration assays rather than a generalized role across all DLBCL variants. Additionally, despite BCL7A’s prevalent expression in the corresponding tumor types, its functional influence appears to be context-dependent, suggesting variability in its role across different cellular environments.

The identification of the BCL7A arginine anchor enhances our understanding of how human SWI/SNF complexes achieve precise chromatin regulation. Together with SMARCB1, BCL7A forms a dual arginine anchor system that ensures robust and context-specific nucleosome binding. Our studies suggest that the BCL7A N-terminal and SMARCB1 C-terminal arginine anchors alternate their engagement with the same nucleosome acidic patch, thereby stabilizing nucleosome binding (Fig. 1B). This dual anchoring system is crucial for the accurate positioning and activity of the human SWI/SNF complexes on chromatin, which in turn facilitates efficient histone displacement (Fig. 2B and 2C) and transcriptional regulation (Fig. 5A and 5D). Disruption of the BCL7A anchor impairs this coordinated mechanism in DLBCL, resulting in defective chromatin remodeling and aberrant gene expression (Fig. 6A and 6J). This dual anchoring mechanism highlights the evolutionary importance of non-catalytic subunits in fine-tuning chromatin remodeling activity and underscores the potential therapeutic implications of targeting these interactions in cancer. Future studies should explore the broader roles of BCL7A in chromatin regulation and its potential as a therapeutic target in malignancies driven by human SWI/SNF complex dysfunction.

In conducting an in-depth functional analysis that is informed by crucial structural insights, we observed that the recent study delineated the structural basis of the BCL7A N-terminus interaction with the nucleosomal acidic patch (Martin et al., 2025). Their findings indicate that BCL7A binds to the acidic patch via residues R4, R7, and R11. This mode of interaction aligns remarkably with our observations, thus providing significant validation for the accuracy of our study and its conclusions.

Interestingly, in our AlphaFold 3-predicted model of the cBAF subcomplex bound to a nucleosome, the HSA domain of BRG1 exhibits a trajectory distinct from that observed in the full cBAF-nucleosome structure (Fig. S1A). Due to the size limitations imposed by the AlphaFold server, we maximized the input of relevant residues, yet we were unable to compare this with the equivalent BCL7A-containing full cBAF complex with nucleosomes. It remains unclear whether this unique structural orientation results from the absence of parts of the base module or if it represents a BCL7A-anchored specific configuration of cBAF. Considering its cooperativity with the SMARCB1 CTD, we believe that this conformation is functionally relevant, presenting an intriguing avenue for future structural investigations.

Rtt102 is recognized as the yeast homolog of BCL7A (Candela-Ferre et al., 2024), and their functional roles reveal both similarities and differences that shed light on the evolutionary development of SWI/SNF complexes. In yeast, Rtt102 forms a complex with Arp7 and Arp9, facilitated by a structurally similar beta-sheet, and co-localizes with the HSA domain of Snf2 or Sth1 (Han et al., 2020; Ye et al., 2019). This interaction enhances the stability of the ARP module, crucial for the chromatin remodeling functions of both SWI/SNF and RSC complexes (Dutta et al., 2017; Lee et al., 2004; Turegun et al., 2013). In contrast, BCL7A in higher eukaryotes employs a positively charged N-terminal α-helix to engage with the nucleosome acidic patch, a feature absent in Rtt102 whose N-terminal α-helix is shorter and devoid of positively charged residues like arginine and lysine (Schubert et al., 2013). Consequently, Rtt102 does not appear to serve as a nucleosome anchor but rather acts predominantly as a structural stabilizer. This functional divergence underscores a significant evolutionary adaptation, highlighting how SWI/SNF remodeling complexes interact with nucleosomes differently in yeast compared with humans. The lack of a nucleosome-binding arginine anchor in Rtt102 emphasizes the evolutionary refinement of BCL7A to assume a more direct role in chromatin remodeling in higher eukaryotes.

BCL7A represents an evolutionarily conserved constituent of the three primary human SWI/SNF chromatin remodeling complexes—cBAF, PBAF, and ncBAF—where it engages with the HSA domain of BRG1 in concert with ACTB and ACTL6A. Currently available experimental data provide no indication that BCL7A’s incorporation into these complexes is influenced by the distinct subunit composition of each assembly, suggesting a consistent mode of interaction across all three variants. cBAF, the most prevalent and canonical variant of the hSWI/SNF complexes, is shown to be the primary association point for BCL7A, as evidenced by BRG1-IP and glycerol gradient sedimentation analyses (Mashtalir et al., 2018). This predominance is further corroborated by V5-FLAG tandem purification experiments conducted on hSWI/SNF complexes from HEK293T cells, which demonstrate lower yields of ncBAF compared with cBAF. Moreover, knockout studies by Sandra Schick et al. (2019) indicate that BCL7A does not exhibit a significant functional preference among the three complexes. Consequently, analyzing BCL7A’s *in vitro* activities within the cBAF complex offers a representative and functionally pertinent model for elucidating its role in chromatin remodeling. Nonetheless, the unique subunit compositions of cBAF, PBAF, and ncBAF likely endow each complex with distinct chromatin remodeling capabilities, genomic localization patterns, and regulatory functions. Notably, PBAF is distinguished by the inclusion of PBRM1 and PHF10, which possess bromodomains and PHD finger domains potentially enhancing nucleosome binding specificity and affinity. In contrast, ncBAF’s lack of SMARCB1 uniquely positions BCL7A as the sole non-­catalytic subunit capable of interacting with the nucleosome acidic patch, possibly facilitating non-competitive nucleosome binding and influencing remodeling activity. Additionally, single-cell perturbation screens suggest a more defined functional relationship between BCL7A and the components of the ncBAF complex (Otto et al., 2023), underscoring a potentially unique role for BCL7A within this assembly. These observations underscore the necessity of exploring BCL7A’s functions within the PBAF and ncBAF complexes in the future, as its role may vary significantly depending on the specific hSWI/SNF complex and the biological context.

BCL7A’s selective regulation of approximately 200 target genes, a subset distinct from the broader gene repertoire commonly influenced by the hSWI/SNF complexes, indicates a specialized role facilitated by potentially rare subcomplexes rather than the entire hSWI/SNF machinery. This specificity suggests that the use of BRG1 knockdown experiments to explore BCL7A’s functions might lead to misleading results due to the wide-ranging, nonspecific effects of BRG1 depletion, which contrasts sharply with the precise regulatory influence of BCL7A. This distinction highlights BCL7A’s mechanistic autonomy in chromatin remodeling within the broader framework of the human SWI/SNF complexes. As a core component present in all known human SWI/SNF variants (cBAF, PBAF, and ncBAF), BCL7A appears to perform both common and variant-specific roles. Structural and biochemical investigations confirm its critical involvement in nucleosome interaction, a fundamental aspect presumably central to its chromatin remodeling capabilities. The enrichment of BCL7A at specific promoters may indicate a targeted biological preference or variant-driven recruitment strategy. Although the precise genomic binding profiles for different hSWI/SNF subtypes remain undefined, our results confirm that the arginine anchor of BCL7A is crucial for its function across all genomic contexts. This study primarily addresses the basic biochemical interactions of BCL7A with nucleosomes, leaving the exploration of its specific interactions with different hSWI/SNF variants and their consequent regulatory outcomes for subsequent research. Future studies, particularly those employing variant-specific knockdown strategies, are essential to delineate these variant-dependent functions. However, our current findings lay a foundational understanding of the mechanistic principles governing BCL7A’s role in chromatin remodeling across all associated complexes.

## Supplementary Material

pwaf114_Supplementary_Data

## Data Availability

Supplementary data are available online. The raw high-throughput sequencing data from RNA-seq, ATAC-seq, and CUT&Tag experiments have been deposited in the Gene Expression Omnibus (GEO) under the following accession numbers: GSE296461, GSE296462, and GSE296463. The mass spectrometry data are available in the PRIDE repository (ebi.ac.uk/pride/login) under Project accession: PXD064944. The structural model generated using AlphaFold3 has been deposited in the ModelArchive database (modelarchive.org/doi/10.5452/ma-22h5b).

## Abbreviations

ARP, actin-related protein; BCL7A, B-cell CLL/lymphoma 7 protein family member A; cBAF, canonical BRG1/BRM-­associated factor; DLBCL, diffuse large B-cell lymphoma; HSA, helicase-SANT-associated domain; hSWI/SNF, human switch/sucrose non-fermentable; ncBAF, non-­canonical BRG1/BRM-associated factor; NCP, nucleosome core particle; PAE, predicted alignment error; PBAF, ­polybromo-associated BRG1/BRM-associated factor; pLDDT, predicted local distance difference test

## Code availability

The scripts used to analyze the sequencing data and structure prediction data from this study are available at Zenodo (doi.org/10.5281/zenodo.15619095).
