# Supplementary material for: BCL7A’s arginine anchor links nucleosome recognition to chromatin remodeling and diffuse large B-cell lymphoma tumor suppression
Source: Protein Cell. 2026 Jan 2;17(5):452–70. doi: 10.1093/procel/pwaf114 (PMC13161475; doi:10.1093/procel/pwaf114)
Supplement: pwaf114_Supplementary_Data [file pwaf114_supplementary_data.pdf]

BCL7A's Arginine Anchor Links Nucleosome Recognition to Chromatin Remodeling and DLBCL Tumor Suppression

Jingdong Xue[1], Kai Tian[2], Xiang Xu[3], Yuqian Feng[1], Ming Yu[1], Min Hao[1], Mingqian Hu[1], Wenhan Wang[4], Jiao Ma[4], Yixuan Pan[1], Mengyuan Peng[1], Jun Wu[3,5,6], Shuang He[7], Xizi Chen[7], Yanhui Xu[7], Wenjuan Wang[8,*], Yimin Lao[1,*] and Bing Li[1,*]

[1] Department of Biochemistry and Molecular Cell Biology, Key Laboratory of Cell Differentiation and Apoptosis of Chinese Ministry of Education, Shanghai Key Laboratory for Tumor Microenvironment and Inflammation, Shanghai Jiao Tong University School of Medicine, Shanghai, China.

[2] Department of Orthopedic Oncology, Shanghai Bone Tumor Institute, Shanghai General Hospital, Shanghai Jiao Tong University School of Medicine, Shanghai, China.

[3] Department of Laboratory Medicine, Jiading Branch of Shanghai General Hospital, Shanghai Jiao Tong University School of Medicine, Shanghai, China.

[4] Department of Biochemistry and Molecular Cell Biology, Shanghai Jiao Tong University School of Medicine, Shanghai, PR China.

[5] Clinicopathological Diagnosis & Research Center, The Affiliated Hospital of Youjiang Medical University for Nationalities, Baise, China.

[6] Key Laboratory of Tumor Molecular Pathology of Guangxi Higher Education Institutes, Baise, China.

[7] Fudan University Shanghai Cancer Center, Institutes of Biomedical Sciences, New Cornerstone Science Laboratory, State Key Laboratory of Genetics and Development of Complex Phenotypes, Department of Biochemistry and Biophysics, School of Life Sciences, Shanghai Key Laboratory of Radiation Oncology and Shanghai Key Laboratory of Medical Epigenetics, Shanghai Medical College of Fudan University, Shanghai, China.

[8] Reproduction Medical Center, Xinhua Hospital affiliated to Shanghai Jiao Tong University School of Medicine, Shanghai, China.

* Correspondence: Wenjuan Wang (sdwangwj@126.com); Yimin Lao (laoyimin@sjtu.edu.cn); Bing Li (bingli@shsmu.edu.cn)

SUPPLEMENTARY INFORMATION

I. Material and Methods:

Plasmid construction

The cDNA of BCL7A was sourced from the DNA core library at SHSMU. Truncations and point mutations of BCL7A were introduced using PCR or overlapping PCR techniques, employing primer pairs designed with specific mutations. For overexpression in HEK293T cells, the cDNA of BCL7A and its variants were cloned into the pCDH-puro-CMV (Supplemental Table 1, #pMD117) and pCDH-EGFP-CMV (#pMD303) lentiviral expression vectors using 5'-SalI and 3'-NotI restriction sites. To construct a C-terminus truncation of SMARCB1 in HEK293T cells, a guide sequence targeting exon 8 of SMARCB1 was inserted into the pSpCas9(BB)-2A-puro (#pMD264) plasmid using BbsI restriction sites.

Mammalian cell culture and lentiviral infection

The OCI-LY1 diffuse large B-cell lymphoma cell line (Supplemental Table 4) was cultured in IMDM medium with 20% fetal bovine serum (FBS), while HEK293T cells were maintained in DMEM medium supplemented with 10% FBS. Both cell lines were incubated at 37 °C in a humidified 5% CO_2 atmosphere. Periodic PCR testing for mycoplasma using specific primers Myco-F (Supplemental Table 2, Primer #8788) and Myco-R (Primer #8789) confirmed the absence of contamination in the cultures. Cell viability was assessed using the Thermo Countess, requiring a minimum of 90% live cells for OCI-LY1 and 95% for adherent cells to proceed with experiments.

BCL7A and its variants were stably expressed in OCI-LY1 cells through lentiviral transduction. HEK293T

cells were transfected with a plasmid mixture (pMD2.G: pMDLg/pRRE: pRSV-Rev: pCDH vector = 3:5:2:5), where the pCDH vector included a GFP sequence under the EF-1α promoter for cell sorting. At 48 hours post-transfection, the medium was collected, filtered through a 0.45 μm membrane, and concentrated using a TAKARA Lenti-X virus concentrator. The viral titer was determined using the LV900 virus quantification kit. For infection, 2×10^5 OCI-LY1 cells were seeded in a 24-well plate, treated with the viral concentrate, sealed with parafilm, and centrifuged at 600 × g for 90 minutes at 37 °C. After 24 hours, the medium was refreshed. Once the cell count reached 2×10^6, GFP-positive cells were isolated by flow cytometry. Successful expression of BCL7A and its variants was verified by Western blot analysis.

Nuclear extract isolation and Western blot

For nuclear extraction, cells were resuspended in Hypotonic Buffer (10 mM Tris-HCl pH 7.5, 10 mM KCl, 1.5 mM $MgCl_2$, 1 mM DTT, 1 mM PMSF, protease inhibitor cocktail) and incubated on ice for 10 minutes. Cells were then centrifuged at 3000 rpm for 20 minutes at 4°C and resuspended in High-Salt Buffer (50 mM Tris-HCl pH 7.5, 300 mM KCl, 1 mM $MgCl_2$, 1 mM EDTA, 1% NP-40, 1 mM DTT, 1 mM PMSF, protease inhibitor cocktail). Following a 2-hour incubation at 4°C, the lysate was centrifuged at 13000 rpm for 60 minutes, and the supernatant was collected as nuclear extract.

For protein analysis, the nuclear extract was mixed with 5× SDS-Loading Buffer (300 mM Tris-HCl pH 8.0, 10% SDS, 20 mM EDTA, 25 mM βME, 0.1% bromophenol blue, 50% glycerol) in a 4:1 ratio and heated at 98°C for 10 minutes. The sample was then electrophoresed on an 8% or 10% SDS-PAGE gel and transferred to an NC membrane. The membrane was blocked with 10% milk in TBST for 30 minutes at room temperature, followed by overnight incubation with the primary antibody in 2% milk at 4°C or for 2 hours at room temperature. After two washes in 2% milk, the membrane was incubated with the secondary antibody in 2% milk at room temperature for 45 minutes, then washed twice with 2% milk and three times with TBST. Detection was performed using the Bio-Rad ChemiDoc chemi channel after applying the ECL solution. All primary antibodies used are specified below, where applicable, and were used at the following dilutions: ARID1A (Abclonal A16648):1:3000, ACTB (Abclonal AC004):1:3000, BAF155:1:3000, BCL7A:1:2000, BRD7 (Abclonal A2308):1:3000, BRD9 (Abclonal A7133):1:3000, BRG1 (Abclonal A2117):1:3000, SMARCB1 (CST D8M1X):1:5000, V5 (Abclonal

AE017):1:3000, FLAG-HRP (Sigma A8592):1:5000.

Competition cell growth assay

To assess the growth rate of diffuse large B-cell lymphoma (DLBCL) cells under various conditions, a modified competitive growth assay based on Gavira et al. (Baliñas-Gavira et al., 2020) was utilized. OCI-LY1 cells, stably expressing either wild-type or mutant BCL7A, were produced through lentiviral infection and GFP-expressing cells were isolated using flow cytometry to ensure uniform GFP expression. Parental and GFP-expressing OCI-LY1 cells (10^5 each) were co-cultured in 12-well plates at 37°C. Cells were resuspended, diluted, and refreshed with new medium every 48 hours to promote optimal growth. At specific time points, flow cytometry was employed to measure the proportion of GFP-positive cells within the live cell population, with values normalized to initial counts to calculate growth ratios. Each condition was tested in triplicate across three independent experiments. Statistical analysis was performed using an unpaired Student's t-test.

RNA isolation and RT-qPCR

Total RNA was isolated from 2×10^6 fresh OCI-LY1 cells. Cells were collected by centrifugation at 1500 rpm for 3 minutes, and the supernatant was discarded. The cell pellet was washed with cold DEPC-treated PBS and lysed in 1 mL of Trizol reagent. The lysate was transferred to a 1.5 mL RNase-free centrifuge tube, mixed with 200 μL of chloroform, and centrifuged at 13,000 rpm for 30 minutes at 4 °C. The RNA was then precipitated from the aqueous phase using isopropanol, washed with 75% ethanol, and resuspended in RNase-free water. RNA concentration and purity were assessed using a NanoDrop spectrophotometer.

RT-qPCR reactions were prepared in a 384-well plate, with each 10 μL reaction containing Realab Green PCR Fast Mix (Beijing LABLEAD lnc.), cDNA template, forward and reverse primers, and nuclease-free water. The plate was sealed with optical adhesive film and amplified using a Roche LightCycler 480. The cycling conditions were: 95 °C for 10 minutes, followed by 40 cycles of 95 °C for 15 seconds and 60 °C for 1 minute. Post-amplification, a melting curve analysis was performed by increasing the temperature from 65 °C to 95 °C while monitoring fluorescence. Data were analyzed using LightCycler 480 software, and relative gene expression

levels were calculated using the $2^{-\Delta\Delta Ct}$ method, normalized against the housekeeping gene *GAPDH*.

RNA-seq

Sequencing libraries were prepared from 1 µg of total RNA using the Abclonal Fast RNA-seq Library Prep Kit, followed by sequencing on the Illumina NovaSeq 6000 platform via Novogene. Raw RNA-seq data were processed using MobaXterm software. Quality control was conducted with FastQC, removing adapter sequences and low-quality reads. The high-quality reads were aligned to the GRCh38 (hg38) reference genome using the STAR aligner (Dobin et al., 2013) with default parameters. PCR duplicates were removed using Samtools rmdup (Li et al., 2009) with standard settings. Gene expression was quantified using the subread featureCounts tool (Liao et al., 2014), and differential expression analysis was performed using the limma (Ritchie et al., 2015) and edgeR (Robinson et al., 2010) packages in RStudio. Differentially expressed genes (DEGs) were identified with criteria of fold change > 1.25 or < 0.8, and p-value < 0.05, with adjustment for multiple testing. Gene Ontology (GO) enrichment analysis was conducted using the clusterProfiler package in R, highlighting the top three significantly enriched GO terms for each category.

CUT&Tag

Fresh cells (1×10^5) were processed using the Vazyme Hyperactive Universal CUT&Tag Assay Kit for Illumina (TD903) according to the manufacturer's standard protocol. Each sample was treated with 1 µL of V5 antibody (CST 13202S) and 0.5 µL of secondary antibody. The resulting DNA library was purified using agarose gel electrophoresis and assessed for quality with the Bioanalyzer. Sequencing was conducted on the NovaSeq X Plus platform via Novogene sequencing services. Raw sequencing data were subjected to quality control with FastQC, where adapter sequences and low-quality reads were removed. High-quality reads were aligned to the GRCh38_p13 reference genome using Bowtie2 (Langmead and Salzberg, 2012) with default parameters. PCR duplicates were removed using Samtools rmdup. Significant peaks were identified using the DiffBind package in RStudio by comparing experimental groups to the control group, with a significance threshold of p-value < 0.05. Peak profiles were computed using computeMatrix, and peak calling was performed using SEACR (Meers et al.,

2019). The bigWig files were generated using bamCoverage (Ramírez et al., 2016) from the deepTools suite and visualized in IGV. Chromatin feature annotation was performed using the HOMER module (Heinz et al., 2010) to analyze the genomic context of the identified peaks.

ATAC-seq

A total of 1×10^5 cells were washed with cold PBS and subsequently with ATAC-wash buffer (10 mM Tris-HCl pH 7.4, 10 mM NaCl, 3 mM $MgCl_2$, 0.1% Tween-20). Cells were lysed using ATAC-lysis buffer (10 mM Tris-HCl pH 7.4, 10 mM NaCl, 3 mM $MgCl_2$, 0.1% Tween-20, 0.01% IGEPAL CA-630, 0.1% Digitonin) for 10 minutes on ice. Following lysis, cells were washed again with ATAC-wash buffer and centrifuged to remove the supernatant. Tagmentation was conducted using the Vazyme TruePrep DNA Library Prep Kit V2 for Illumina (TD501) at 37°C for 30 minutes. The DNA was then amplified using NEB Next High-Fidelity 2× PCR Master Mix and the resulting library was purified via agarose gel electrophoresis, selecting fragments between 200 bp and 700 bp. Quality control of the purified library was performed using the Bioanalyzer. Sequencing was carried out on the NovaSeq platform by ANOROAD. Raw sequencing data were processed with FASTQC to remove adapter sequences and low-quality reads. High-quality reads were aligned to the hg38 reference genome using STAR. Peak calling was performed using MACS2 (Zhang et al., 2008) with default parameters. The bamCoverage tool from the deepTools suite generated bigWig files, which were visualized in IGV.

Tumor formation

Immunodeficient nude mice (BALB/c-nu, athymic) were utilized for tumorigenesis studies. The OCI-LY1 cell line, engineered to stably express BCL7A-related genes, was employed for tumor induction. Cells were centrifuged at 3000 rpm for 3 minutes, resuspended in fresh IMDM medium, and counted. A total of 5×10^6 cells were prepared by centrifugation and resuspended in 500 μL of IMDM. The cell suspension was cooled on ice in a microcentrifuge tube. Thawed Corning Matrigel was added to the cells in a 1:1 ratio, and the mixture was gently pipetted for homogenization. Prior to injection, mice were prepped by cleaning the thoracic and axillary areas with alcohol swabs. Using a 1 mL syringe, 200 μL of the cell-Matrigel mixture was subcutaneously injected into

the axillary region of each mouse. Five mice were inoculated per tumor cell type, with one injection per mouse. Mice were grouped by cell type, identified by toe clipping, and the inoculation day was recorded as day 0. Tumor growth was monitored on days 7, 14, 21, and 28 by measuring body weight and tumor dimensions (length L and width W) using calipers. Tumor volume (V) was calculated using the formula: $V = 0.5 \times L \times W^2$. On day 28, mice were euthanized post body weight and tumor volume assessment, tumors were excised and weighed, and samples were processed for histological analysis. A double-blind method was applied to the analysis to ensure unbiased evaluation, with randomly selected samples counted and ratios calculated.

CRISPR-Cas9 Editing

Two guide RNAs (gRNAs) targeting exon 8 of SMARCB1 were designed using the Benchling CRISPR Guide RNA Design Tool (https://www.benchling.com/crispr). The sequences for the gRNA were: gRNA-1: 5'-CATCCGGAACACGGGCGATG-3' and gRNA-2: 5'-ACACGGGCGATGCGGACCAG-3'. Each gRNA was cloned into the pSpCas9(BB)-2A-puro vector using BbsI sites. Equal amounts of the plasmids, each harboring one of the gRNAs, were co-transfected into HEK293T cells using Lipo3000 transfection reagent. Cells were selected with 2 μg/mL puromycin between 36- and 72-hours post-transfection. Following selection, cells were counted, diluted, and plated at one cell per well in a 96-well plate to isolate single clones. The expression of SMARCB1 in these clones was analyzed by Western blot to detect clones with reduced SMARCB1 protein size. Genomic DNA was extracted from selected clones, and the targeted region of exon 8 was PCR-amplified and sequenced to verify gene editing. Additionally, RNA was extracted, reverse-transcribed, and the SMARCB1 cDNA was amplified and sequenced to assess transcript-level mutations.

BAF complex purification

Nuclear chromatin remodeling complexes were isolated from HEK293T cells stably expressing FLAG-DPF2 and V5-BCL7A through sequential immunoprecipitation using V5 and FLAG affinity beads (Supplemental Table 5). These HEK293T stable cell lines were established by two rounds of lentiviral transduction. Adherent cells were trypsinized, resuspended in serum-free medium, and cultured in glass spinner flasks at 37°C and 8% CO_2,

with agitation at 170 rpm. Cell density was monitored regularly, and medium was replenished as necessary. Upon reaching a total numberof 10^9 cells, they were collected for nuclear protein extraction.

For nuclear extraction, cells were lysed in Hypotonic Buffer (10 mM Tris-HCl pH 7.5, 10 mM KCl, 1.5 mM $MgCl_2$, 1 mM DTT, 1 mM PMSF, protease inhibitor cocktail), incubated on ice for 10 minutes, centrifuged at 3000 rpm for 20 minutes at 4°C, and then resuspended in High-Salt Buffer (50 mM Tris-HCl pH 7.5, 300 mM KCl, 1 mM $MgCl_2$, 1 mM EDTA, 1% NP-40, 1 mM DTT, 1 mM PMSF, protease inhibitor cocktail). After a 2-hour incubation at 4°C, the lysate was centrifuged at 13000 rpm for 60 minutes. The supernatant was used for cBAF complex purification via V5-FLAG tandem purification.

V5 affinity purification involved pre-equilibrating V5 affinity beads with High-Salt Buffer, incubating them with the nuclear extract on a rotating platform at 4°C for 4 hours, and washing the beads twice with High-Salt Buffer and once with FLAG Elution Buffer (50 mM HEPES pH 7.9, 100 mM NaCl, 2 mM $MgCl_2$, 0.02% NP-40, 10% Glycerol, 1 mM PMSF, 1 mM Benzamidine). Elution was achieved by incubating the beads with FLAG Elution Buffer containing 0.5 mg/mL V5 peptide for 1 hour, followed by a second 30-minute elution. Eluates were combined, cleared of beads using a gravity column, and collected. FLAG affinity purification followed, with anti-DYKDDDDK affinity beads (Smart-Lifesciences, PP100378) washed and incubated with the V5 eluate for 4 hours at 4°C, washed once with FLAG Elution Buffer, and eluted using FLAG Elution Buffer containing 0.3 mg/mL FLAG peptide for 1 hour, followed by a second 30-minute elution. Eluates were combined, cleared of beads, and the purified complex was concentrated using a 30 kDa ultrafiltration centrifugal filter.

The purified complexes were analyzed by SDS-PAGE with Coomassie blue staining. Equal volumes of each purified complex sample, along with a BSA concentration standard series, were resolved on 8% SDS-polyacrylamide gels. Protein concentrations were quantified by densitometric analysis, comparing band intensities of either ARID1A or BRG1 against the BSA standard curve. The final product was aliquoted, snap-frozen in liquid nitrogen, and stored at –80°C for future use.

Nucleosome reconstitution

The nucleosome reconstitution was conducted with minor modifications to previously described methods (Lee et al., 2013). The 45N45-DNA was purified from a PCR product using plasmid pGEM-3Z/601 reverse

(#pBL386) as the template and primers primer-#6300 and primer-#6301 (Supplemental Table 3), employing Taq DNA polymerase (NEB). The 237-bp product was purified with the Bio-Rad 491 DNA purification system following separation by 3.5% native PAGE. Similarly, 216L-DNA was obtained from EcoRV-digested plasmid pBS-216L-16X (#pBL645), and the 216-bp fragments were purified using the same system. Purified core histones from HeLa cells (Li Lab stock) were used to reconstitute nucleosomes with these DNA products.

Nucleosome reconstitution involved mixing core histones and DNA at a 1:1.2 ratio in TEB Buffer (20 mM Tris-HCl pH 7.5, 1 mM EDTA, 1 mM βME) with 2 M NaCl, followed by incubation at 30°C for 2 hours. The mixture was then dialyzed sequentially against decreasing concentrations of NaCl (1.2 M, 1 M, 0.8 M, 0.6 M) at 30°C for 2 hours each, and finally against TEB Buffer at 4°C overnight. Mono-nucleosomes were purified using the Bio-Rad 491 system and 3.5% native PAGE, run in 0.3×TBE Buffer at 20 W for 2 hours at 4°C. The elution buffer was 10 mM Tris-HCl (pH 7.5). Fractions were analyzed by native PAGE and ethidium bromide staining, with nucleosome-containing fractions pooled and concentrated using a 30 KD ultrafilter. The concentrated nucleosome was mixed with Stocking Buffer (250 mM NaCl, 5 mM βME, 50% Glycerol) at a 4:1 ratio, snap-frozen in liquid nitrogen, and stored at -80°C.

Sliding assay

Sliding assays were conducted with minor modifications to previously described methods (Huh et al., 2012). Purified cBAF complexes were thawed on ice and diluted in FLAG-Elution buffer. The reaction mixture, also prepared on ice, consisted of Remodeling Buffer (20 mM Tris-HCl pH 7.6, 50 mM KCl, 5 mM $MgCl_2$, 0.1 mg/mL BSA, 10% glycerol, 1 mM PMSF), supplemented with designated nucleosome, 2 mM ATP, and 3 ng/µL Sink-DNA. The removal mix contained Remodeling Buffer and 30 ng/µL Competitor-DNA. The reaction was initiated by adding the cBAF complex to the mixture to achieve the specified final concentration, followed by mixing and incubation at 30°C for 3 hours. Post-incubation, the removal mix was added, and the mixture was further incubated at 4°C for 1 hour. The samples were then loaded onto a 3.5% native polyacrylamide gel and subjected to electrophoresis in 0.3×TBE buffer at 220 V for 150 minutes at 4°C. The gel was stained with 50 µg/mL ethidium bromide for 1 hour and destained with water at room temperature. DNA visualization was performed using the Bio-Rad ChemiDoc Ethidium-Bromide channel, and nucleosome substrate and free DNA product

intensities were quantified using ImageJ software.

ATPase assay

The ATPase assays were conducted with minor modifications from previously described methods. cBAF complexes were diluted in FLAG-Elution buffer and the reaction mixture was prepared on ice using Remodeling Buffer (20 mM Tris-HCl pH 7.6, 50 mM KCl, 5 mM $MgCl_2$, 0.1 mg/mL BSA, 10% glycerol, 1 mM PMSF), supplemented with 10 nM 45N45 nucleosome and 0.5 mM ATP. The cBAF complex was added to the reaction mixture to achieve the specified final concentration, mixed thoroughly, and incubated at 30°C for 1 hour. ADP production was then immediately quantified using the ADP-Glo Kinase Kit (Promega) in a white-bottom 96-well plate. Luminescence was measured with the Molecular Device iD5 Luminescence channel. Relative ATPase activity was determined by normalizing luminescence readings for each sample to the highest value recorded among all treatment groups in the same experimental batch.

Restriction enzyme accessibility assay

We conducted the restriction enzyme accessibility assay using our established protocol. The reaction mix, prepared on ice, included Remodeling Buffer (20 mM Tris-HCl pH 7.6, 50 mM KCl, 5 mM $MgCl_2$, 0.1 mg/mL BSA, 10% glycerol, 1 mM PMSF), 10 nM 45N45 nucleosome, 0.4 U/μL HhaI and 1 mM ATP. The reaction was initiated by adding the cBAF complex to the mix. The 15 μL reaction mixture was incubated at 30°C for 1 hour. Post-incubation, 45 μL of water containing 5 mM EDTA and 1 ng/μL spike-in DNA was added. The mixture was then combined with 60 μL of PCIAA (Phenol: Chloroform: Isoamyl alcohol = 25:24:1), followed by centrifugation at 12,000 rpm for 15 minutes at 4°C. DNA was extracted from the supernatant via ethanol precipitation and analyzed on a 2% agarose gel stained with GelRed. Electrophoresis was performed in 1× TAE buffer at 180 V for 30 minutes, and the gel was imaged using the Bio-Rad ChemiDoc GelRed channel. DNA bands, including spike-in DNA, 45N45-DNA, and cut DNA, were quantified using Image J. The ratios of substrate to product were normalized to the spike-in DNA. The experiment included three biological replicates, and statistical analysis was performed using an unpaired Student's t-test.

II. Supplemental Figures

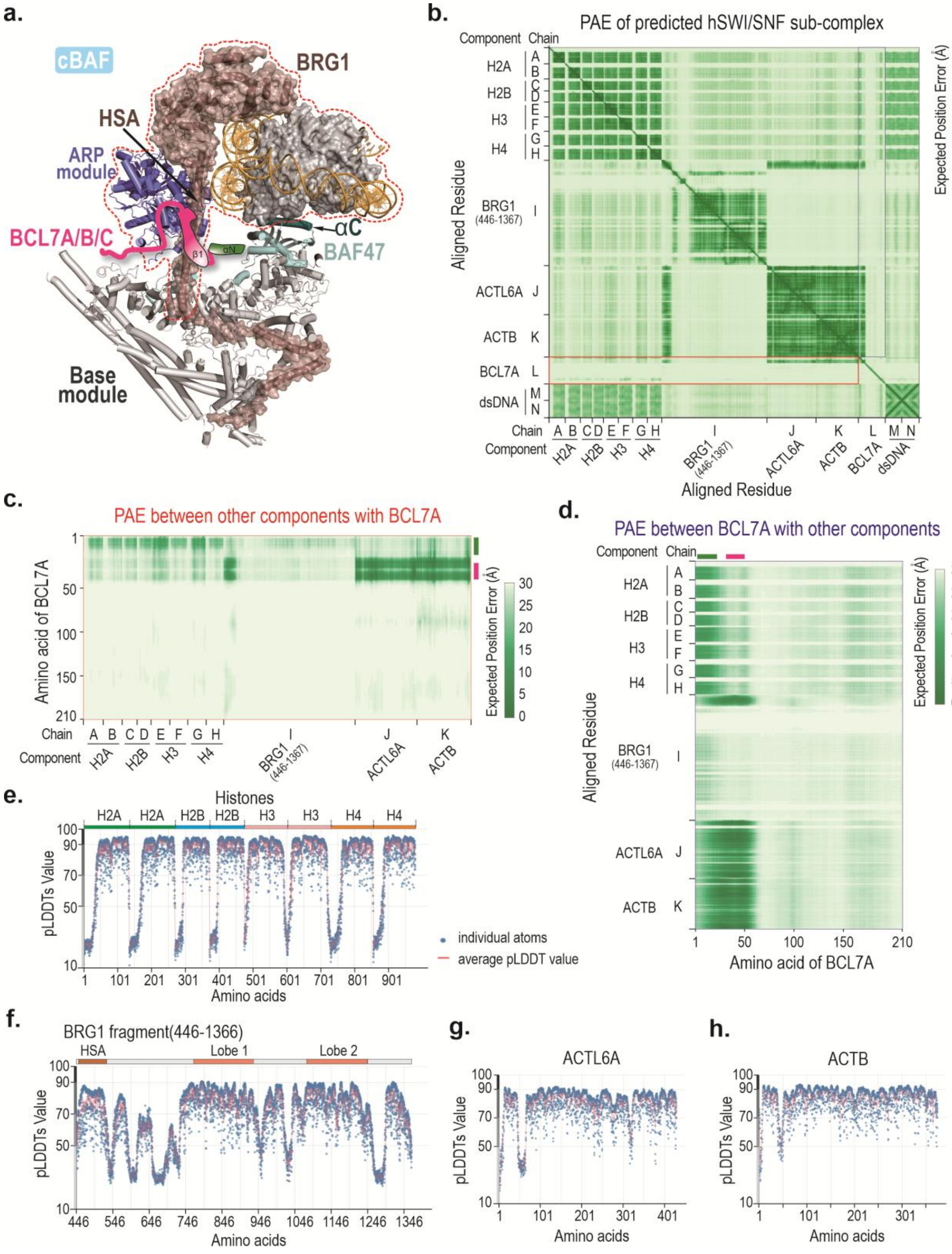

Figure S1. Predicted Structure of the Human SWI/SNF Subcomplex.

(**a**) Predicted localization of BCL7A in the nucleosome-bound cBAF complex (PDB: 6LTJ) from AlphaFold3 analysis shown in Figure 1b, highlighted by a red dashed outline.

(**b**) Predicted Alignment Error (PAE) matrix for predicted human SWI/SNF sub-complex in Figure 1b. The red box highlights BCL7A involving the components of interest in the alignment, while the blue box indicates of those components involving BCL7A.

(**c-d**) The PAE matrix focusing on the interactions between BCL7A and other components of the complex. (c) The PAE values mapping amino acids in BCL7A against histones and other components in the complex, corresponding to the red box in Figure S1b. (d) PAE values mapping the histones and other component in the complex against BCL7A, corresponding to the blue box in Figure S1b. A prominent low-PAE peak near amino acids 1-50 of BCL7A suggests potential interaction sites with these components.

(**e-h**) Predicted local distance difference test (pLDDT) scores for BRG1 fragment (e), histone octamer (f), ACTL6A (g), and ACTB (h) within the predicted hSWI/SNF sub-complex.

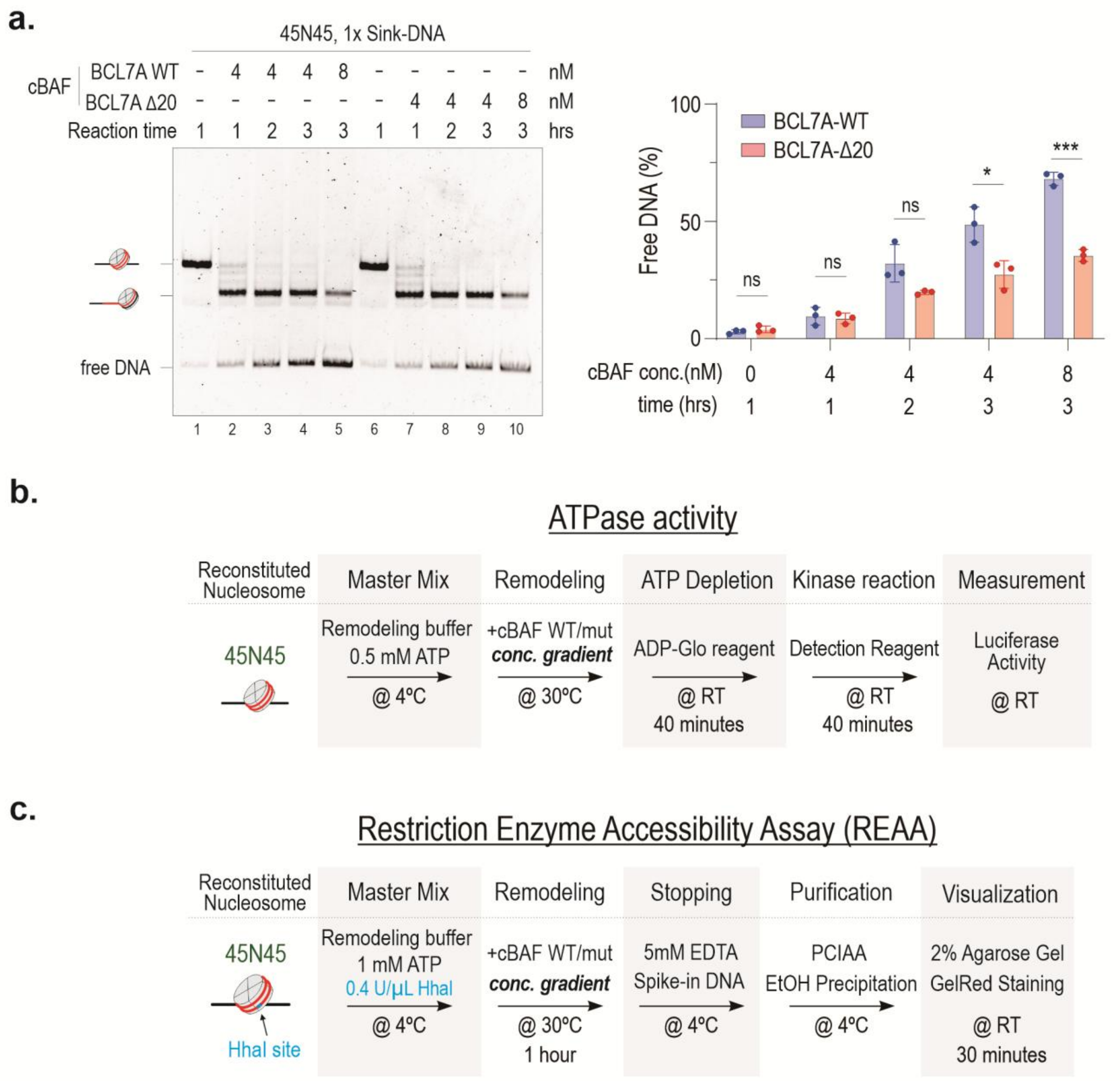

Figure S2. Role of N-terminal α-helix of BCL7A in cBAF Remodeling Activity.

(**a**) Representative sliding assay image of cBAF complexes with 20 nM 45N45 nucleosome (left) and quantification of free DNA production (right), independently validating conclusions from Figure 2b under different experimental conditions. Data show mean ± SD from three individual replicates; *p*-value calculated by unpaired Student's t-test, "ns" indicating non-significance, "*" indicating $p < 0.05$, "***" indicating $p < 0.001$.

(**b**) Workflow of the ATPase assay, related to Figure 2d.

(**c**) Workflow of the restriction enzyme accessibility assay (REAA), related to Figure 2e.

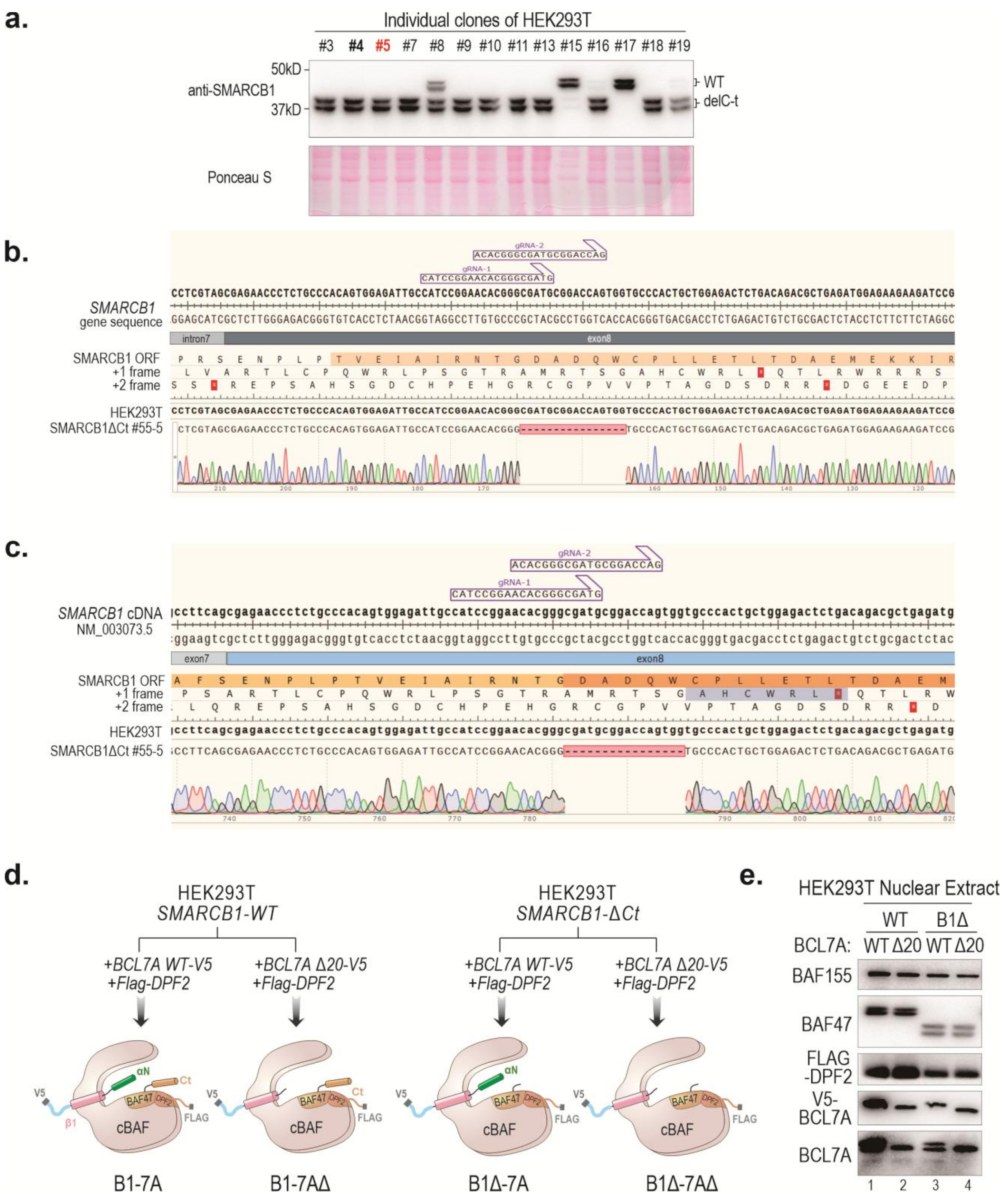

a.
Individual clones of HEK293T
#3 #4 #5 #7 #8 #9 #10 #11 #13 #15 #16 #17 #18 #19
50kD
anti-SMARCB1
37kD
WT
delC-t
Ponceau S
b.
gRNA-2
ACACGGGCGATGCGGACCAG
gRNA-1
CATCCGGAACACGGGCGATG
SMARCB1
gene sequence
intron7
exon8
SMARCB1 ORF
+1 frame
+2 frame
HEK293T
SMARCB1ΔCt #55-5
c.
gRNA-2
ACACGGGCGATGCGGACCAG
gRNA-1
CATCCGGAACACGGGCGATG
SMARCB1 cDNA
NM_003073.5
exon7
exon8
SMARCB1 ORF
+1 frame
+2 frame
HEK293T
SMARCB1ΔCt #55-5
d.
HEK293T
SMARCB1-WT
+BCL7A WT-V5
+Flag-DPF2
+BCL7A Δ20-V5
+Flag-DPF2
HEK293T
SMARCB1-ΔCt
+BCL7A WT-V5
+Flag-DPF2
+BCL7A Δ20-V5
+Flag-DPF2
V5
αN
Ct
BAF47
DPF2
FLAG
β1
cBAF
B1-7A
B1-7AΔ
B1Δ-7A
B1Δ-7AΔ
e.
HEK293T Nuclear Extract
WT
B1Δ
BCL7A: WT Δ20 WT Δ20
BAF155
BAF47
FLAG
-DPF2
V5-
BCL7A
BCL7A
1 2 3 4

Figure S3. Generation of SMARCB1-mutated HEK293T Cell Line and Purification of cBAF Complexes with Distinct Arginine Anchor Configurations.

(**a**) Western blot validation of SMARCB1 C-terminal deletion (ΔCt) in HEK293T clones, highlighting clone #5 with a reduced molecular weight of SMARCB1, which was selected for further experiments.

(**b**) Sanger sequencing of genomic DNA from HEK293T SMARCB1-ΔCt clone #5 confirmed a 16-bp deletion in exon 8 of SMARCB1 (red box), with the corresponding sequence of C-terminal domain highlighted in orange.

(**c**) RT-PCR analysis of SMARCB1 transcripts from clone #5 showing a 16-nt deletion leading to a frameshift and premature stop codon (red TGA), with the resultant C-terminus loss (deep orange box) and 6 amino acids gained (gray box).

(**d**) Strategy for purifying cBAF complexes with specific arginine-anchor mutations in BCL7A and SMARCB1.

(**e**) Western blot analysis of SMARCB1, FLAG-DPF2, and V5-BCL7A expression in wild-type and SMARCB1-ΔCt (B1Δ) HEK293T cells.

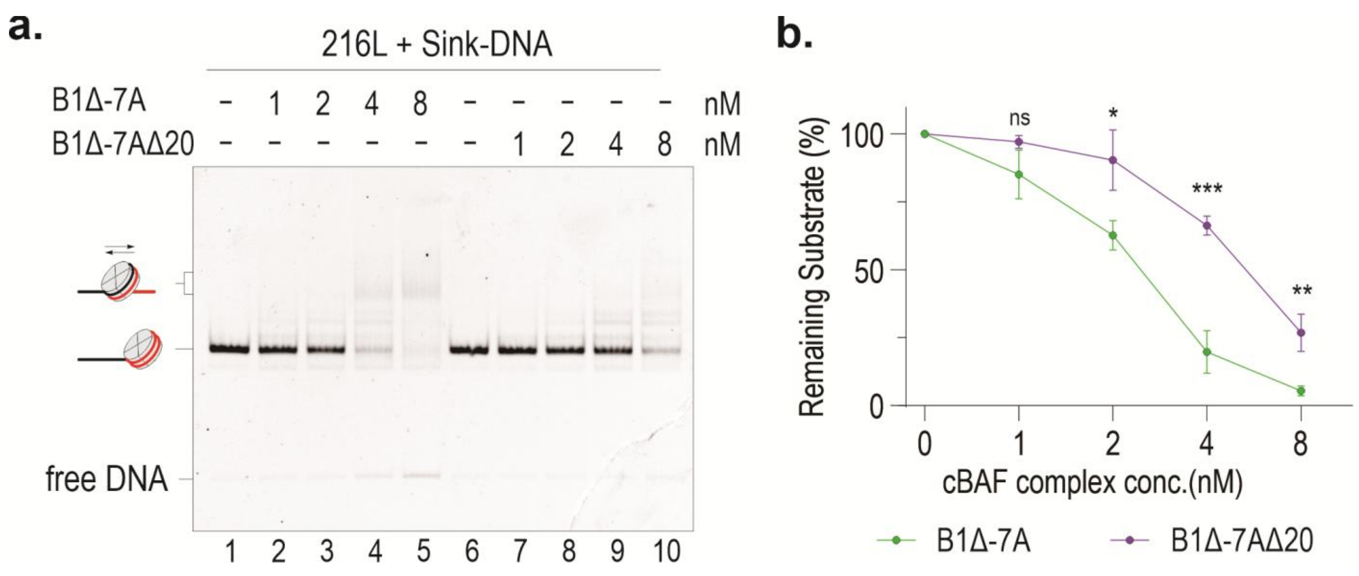

Figure S4. SMARCB1 and BCL7A Work Together in Modulating hSWI/SNF Chromatin Remodeling Activity.

(**a**) Representative image of an *in vitro* sliding assay using specified cBAF complexes with a 20 nM 216L mono-nucleosome.

(**b**) Quantification of remaining substrates in the sliding assay. Data show mean ± SD from three individual replicates; *p*-value calculated by unpaired Student's t-test, "ns" indicating non-significance, "*" indicating $p < 0.05$, "**" indicating $p < 0.01$, "***" indicating $p < 0.001$.

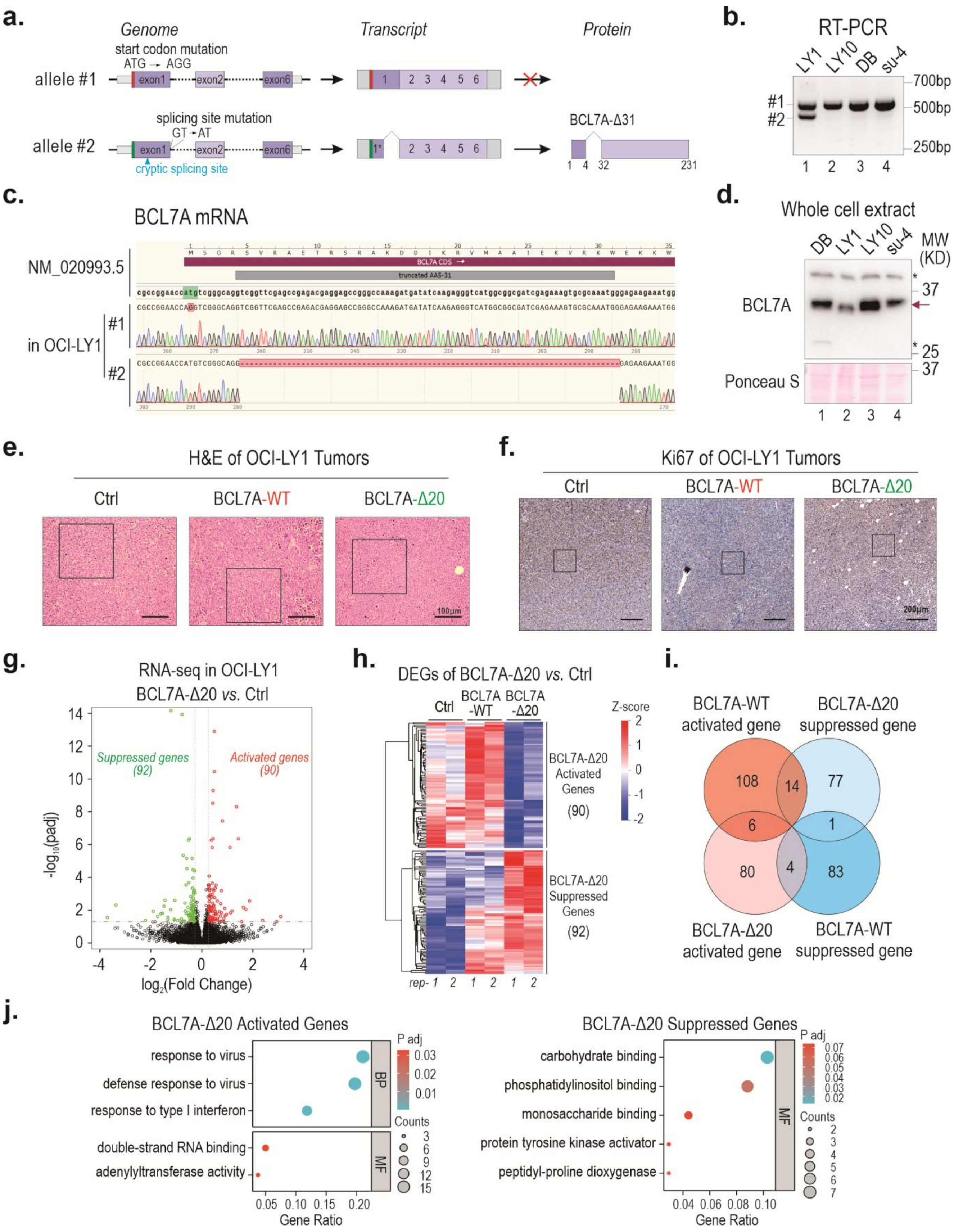
a.
Genome
Transcript
Protein
start codon mutation
ATG → AGG
allele #1
exon1
exon2
exon6
splicing site mutation
GT → AT
allele #2
cryptic splicing site
BCL7A-Δ31
b.
RT-PCR
LY1
LY10
DB
su-4
700bp
500bp
250bp
#1
#2
c.
BCL7A mRNA
NM_020993.5
in OCI-LY1
#1
#2
d.
Whole cell extract
MW (KD)
BCL7A
Ponceau S
37
25
e.
H&E of OCI-LY1 Tumors
Ctrl
BCL7A-WT
BCL7A-Δ20
100μm
f.
Ki67 of OCI-LY1 Tumors
Ctrl
BCL7A-WT
BCL7A-Δ20
200μm
g.
RNA-seq in OCI-LY1
BCL7A-Δ20 vs. Ctrl
Suppressed genes (92)
Activated genes (90)
-log10(padj)
log2(Fold Change)
h.
DEGs of BCL7A-Δ20 vs. Ctrl
Ctrl
BCL7A -WT
BCL7A -Δ20
Z-score
BCL7A-Δ20 Activated Genes (90)
BCL7A-Δ20 Suppressed Genes (92)
rep- 1 2 1 2 1 2
i.
BCL7A-WT activated gene
BCL7A-Δ20 suppressed gene
BCL7A-Δ20 activated gene
BCL7A-WT suppressed gene
108
14
77
6
1
80
4
83
j.
BCL7A-Δ20 Activated Genes
response to virus
defense response to virus
response to type I interferon
double-strand RNA binding
adenylyltransferase activity
BP
MF
P adj
Counts
Gene Ratio
BCL7A-Δ20 Suppressed Genes
carbohydrate binding
phosphatidylinositol binding
monosaccharide binding
protein tyrosine kinase activator
peptidyl-proline dioxygenase
MF
P adj
Counts
Gene Ratio

Figure S5. The N-term α-helix of BCL7A Is Essential for DLBCL Tumor Suppression.

(**a**) Schematic of heterozygous BCL7A mutations in OCI-LY1 cells: one allele with a start codon mutation disrupting translation, and the other with a 5'-splicing site mutation in the first intron, leading to a truncated protein missing amino acids 5–31 due to cryptic splicing.

(**b**) RT-PCR analysis of BCL7A cDNA in DLBCL cell lines showing two bands in OCI-LY1 (wild-type and a shorter isoform) versus single bands in OCI-LY10, DB, and Su-DHL-4.

(**c**) Sequencing of BCL7A cDNA in OCI-LY1 confirms mutations: the longer transcript has a start site mutation impairing translation, and the shorter lacks an 81-nt segment corresponding to amino acids 5-31, resulting in truncation.

(**d**) Western blot analysis of BCL7A in DLBCL cell lines shows reduced protein levels and lower molecular weight in OCI-LY1 (indicated by red arrow), consistent with the truncated transcript.

(**e-f**) Higher-magnification views of the H&E staining (e) and Ki67 immunohistochemical staining (f) of the OCI-LY1 tumors, corresponding to the regions marked in Figure 4j. Insets (black boxes) represent the magnified views displayed in Figure 4j.

(g) Volcano plot of RNA-seq data comparing gene expression between BCL7A-Δ20 expressed and control OCI-LY1 cell lines, with upregulated genes (90) in red and downregulated genes (92) in green.

(h) Heatmap displaying expression differences among BCL7A-Δ20 regulated genes in control, BCL7A-WT and Δ20 OCI-LY1 cells.

(i) Venn diagram shows the overlap of the regulated genes of BCL7A-WT and BCL7A-Δ20.

(j) Top enriched Gene Ontology (GO) terms for the activated genes (right) and suppressed genes (left) by BCL7A-Δ20.

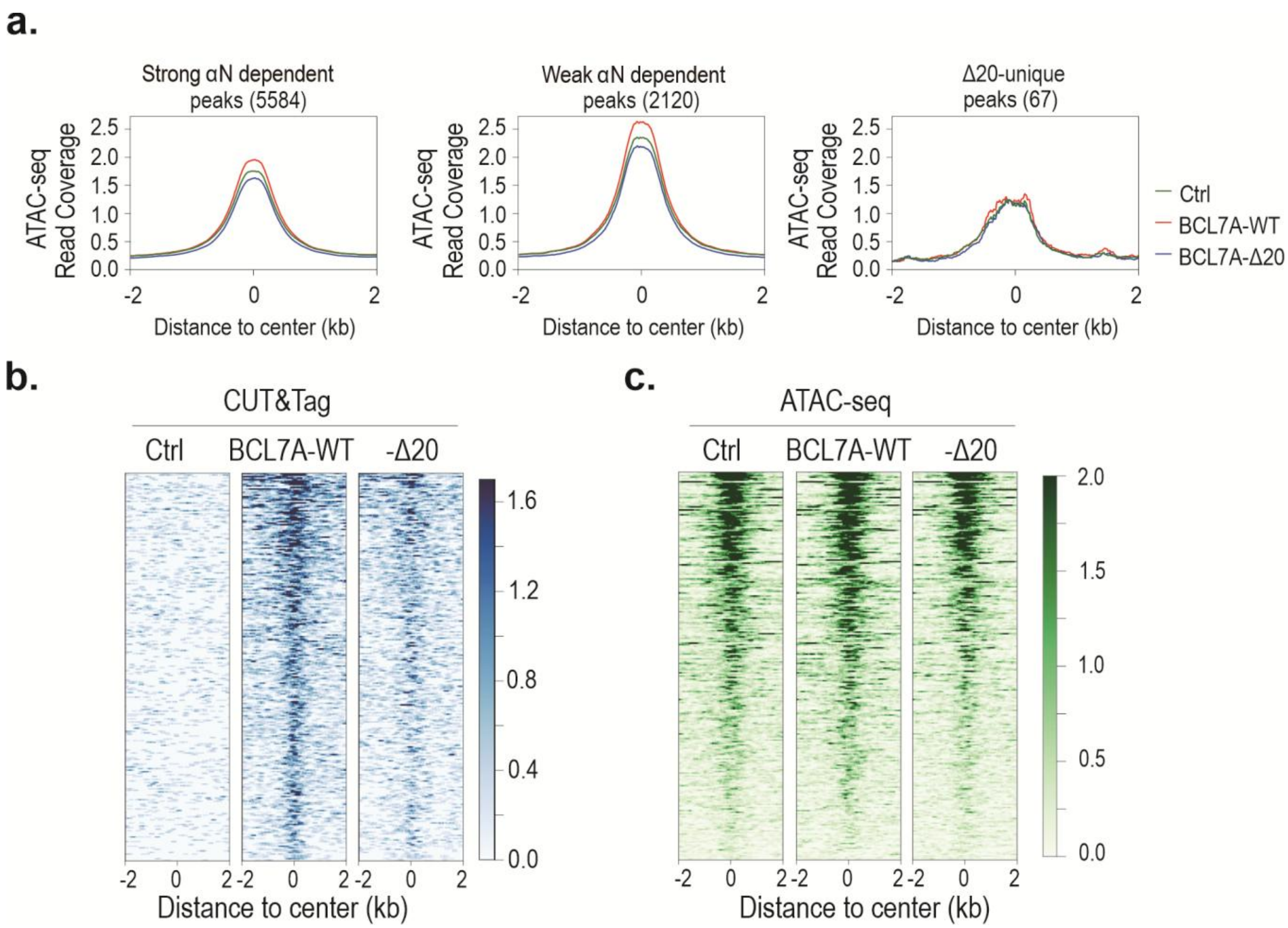

Figure S6. Comprehensive Analysis of BCL7A Binding and Genomic Regulation in OCI-LY1 Cells.

(**a**) Summary metaplots showing accessibility at BCL7A-dependent peaks: strongly dependent (left), weakly dependent (middle), and independent (right) under Ctrl, BCL7A-WT, and BCL7A-Δ20 mutant conditions.

(**b**) Heatmaps depicting BCL7A-WT or Δ20 mutant occupancy near BCL7A-activated genes, corresponding to Figure 6j.

(**c**) Heatmaps of ATAC-seq data showing chromatin accessibility at BCL7A-WT peaks near BCL7A-activated genes for Ctrl, BCL7A-WT, and BCL7A-Δ20 conditions, corresponding to Figure 6k.

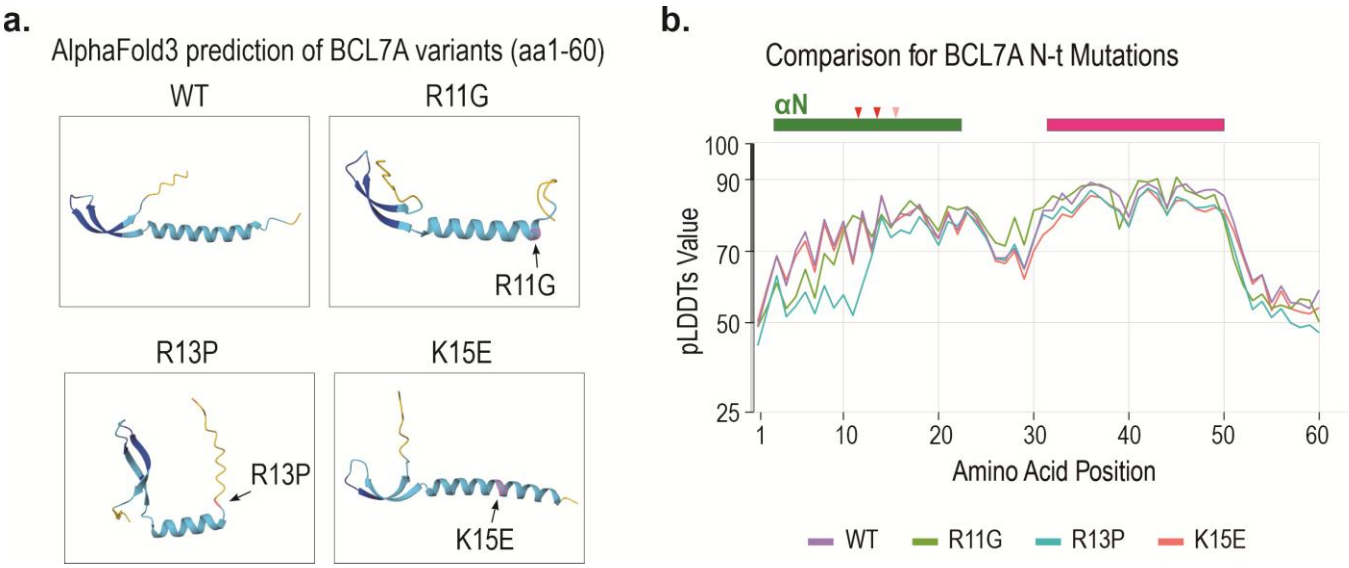

Figure S7. Predicted Structure of BCL7A N-terminal Mutants.

(**a**) Predicted structures of wild-type and mutant (R11G/R13P/K15E) BCL7A residues 1-60 using AlphaFold3.

(**b**) Average pLDDT values for amino acids in various predicted BCL7A variants, highlighting disruptions in α-helical conformation at the N-terminal regions by R11G and R13P mutations compared to wild-type BCL7A.

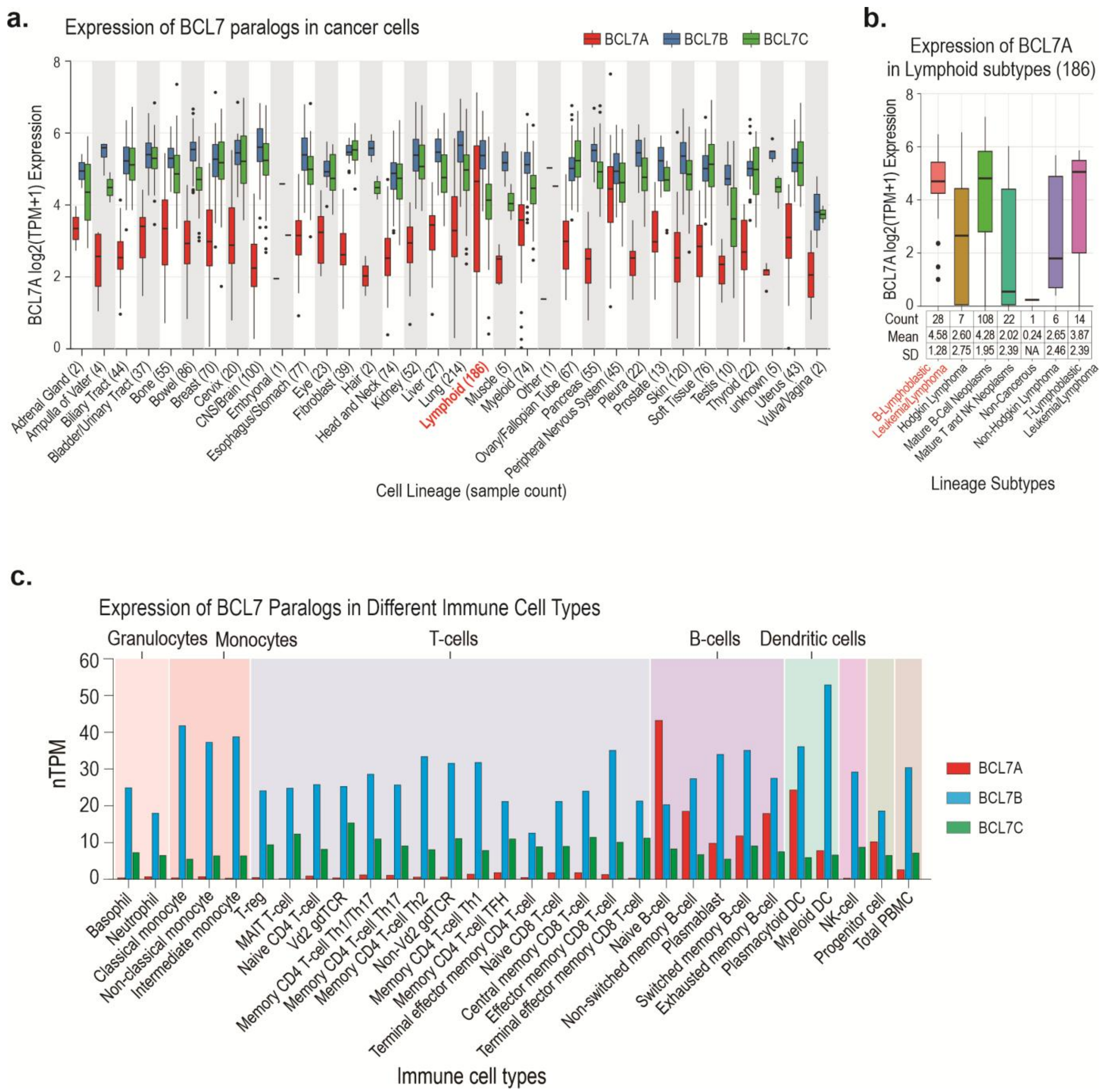

Figure S8. Expression Profiles of BCL7A and Its Paralogs across Malignancies and Normal Tissues.

(**a**) Box plot showing BCL7A, BCL7B, and BCL7C mRNA expression (log2[TPM+1]) across 1,673 cancer cell lines from various lineages (DepMap Public 23Q4), with median and interquartile range (IQR) indicated. Lymphoid lineage is highlighted.

(**b**) Box plot of BCL7A expression across 186 lymphoid malignancy subtypes, indicating median and IQR. B-Lymphoblastic Leukemia/Lymphoma is specifically highlighted.

(**c**) Expression levels of BCL7A, BCL7B, and BCL7C in various human immune cell subsets, sourced from The Human Protein Atlas.

III. Supplemental Tables:

Supplemental Table 1. The list of plasmids used in this study

Plasmids	Backbone	Parental	Plasmid description	Source
#pMD041	/	/	pMD2.G	Li Lab
#pMD044	/	/	pMDLg/pRRE	Li Lab
#pMD045	/	/	pRSV-Rev	Li Lab
#pMD054	pLX304-Blast	/	pLX304-Blast-BCL7A-V5	Li Lab
#pMD117	pCDH-puro-Flag	#pMD117	pCDH-puro-CMV-FLAG-empty	Li Lab
#pMD264	pSpCas9(BB)-2A	/	pSpCas9(BB)-2A-puro-empty	Li Lab
#pMD295	pCDH-puro-Flag	#pMD117	pCDH-puro-Flag-BRD9	Li Lab
#pMD296	pCDH-puro-Flag	#pMD117	pCDH-puro-Flag-DPF2	Li Lab
#pMD297	pCDH-EGFP-V5	#pMD303	pCDH-EGFP-BCL7A-V5	This study
#pMD298	pCDH-EGFP-V5	#pMD303	pCDH-EGFP-BCL7A(del 1-20)-V5	This study
#pMD299	pCDH-EGFP-V5	#pMD303	pCDH-EGFP-BCL7A(del 5-31)-V5	This study
#pMD303	pCDH-EGFP-V5	/	pCDH-EGFP	Li Lab
#pMD311	pSpCas9(BB)-2A	#pMD264	pSpCas9(BB)-BAF47 Ct sgRNA-2	This study
#pMD314	pCDH-EGFP-V5	#pMD303	pCDH-EGFP-BCL7A R11G	This study
#pMD315	pCDH-EGFP-V5	#pMD303	pCDH-EGFP-BCL7A R13P	This study
#pMD316	pCDH-EGFP-V5	#pMD303	pCDH-EGFP-BCL7A K15E	This study
#pMD317	pCDH-EGFP-V5	#pMD303	pCDH-EGFP-BCL7A K19N	This study
#pMD318	pCDH-EGFP-V5	#pMD303	pCDH-EGFP-BCL7A R20K	This study
#pMD319	pCDH-EGFP-V5	#pMD303	pCDH-EGFP-BCL7A R29C	This study
#pMD320	pCDH-EGFP-V5	#pMD303	pCDH-EGFP-BCL7A K30R	This study
#pMD321	pCDH-puro-Flag	#pMD117	pCDH-puro-Flag-BRD7	Li Lab
#pMD333	pCDH-puro-Flag	#pMD117	pCDH-BRG1(HSA-C)	this study
#pBL386	pGEM-T	/	pGEM-3Z/601 reverse	Li Lab(Huh et al., 2012)
#pBL645	pBluescript	/	pBS-216L-16X	Li Lab(Huh et al., 2012)

Supplemental Table 2. The list of primers used in this study

Primers	Name	Sequence
MD829	BCL7A_SalI_F	ACGGTCGACATGTCGGGCAGGTCGGTTCG
MD830	BCL7A_NotI_R	ACGGCGGCCGCCATCTCTTCGGAGTTTTGTTG
5698	BCL7A_1-20del_F	GTTTGTACAAAAAAGTTGGCACCATGGTCATGGCGGCG ATCGAGAAAGTG
5699	BCL7A_5-31del_F	GTACAAAAAAGTTGGCACCATGTCGGGCAGGGAGAAG AAATGGGTGACCGTTGGTG
5700	BCL7A_1-50del_F	GTACAAAAAAGTTGGCACCATGGTGACGGAGCCCAAGG TTGATGAC
5718	BAF47-CtKO-gRNA-1F	CACCGCATCCGGAACACGGGCGATG
5719	BAF47-CtKO-gRNA-1R	AAACCATCGCCCGTGTTCCGGATGC
5720	BAF47-CtKO-gRNA-2F	CACCGACACGGGCGATGCGGACCAG
5721	BAF47-CtKO-gRNA-2R	AAACCTGGTCCGCATCGCCCGTGTC
5722	BAF47-exon8-F	CTCGTAGCGAGAACCCTCTGCC
5723	BAF47-exon-R	CTAGCAGCACTAGAGGTACTCTGTC
6261	BCL7A_genome_F	GAACCGGTGCCTTCAACTCGGAGAAG
6262	BCL7A_R4_F	CTCCCCGCCGGAACCATGTCGGGCAG
6263	BCL7A_exon1_R	GCGCACTTTCTCGATCGCCGCCATGAC
6264	BCL7_intron1_R	CTCGGAGCCTGCACAATGGAGCTC
6265	BCL7A_421R	CAGAAGCATCTTCAGCTCCTGGGTGC
6266	BCL7A_exon1_F	GCGGAGCGCGAGCAGGAC
6274	B7A_mRNA_F1	CACTGGGCCAGGCGCGCGGCGGCCCCGGGCTTTG
6300	BL_45N45_F	AACACTATCCGACTGGCACCGGC
6301	BL_45N45_R	ACACCGAGTTCATCCCTTATGTGATG
6309	BCL7A_R4G_XbaI_F	AGATTCTAGACAAAAAAGTTGGCACCATGTCGGGCGGG TCGGTTCG
6310	BCL7A_mut_XbaI_F	AGATTCTAGACAAAAAAGTTGGCACCATGTCGGGCAGG TCGGTTCGAGCC
6311	BCL7A_R11G_F	GGCAGGTCGGTTCGAGCCGAGACGGGGAGCCGGGCCA AAG
6312	BCL7A_R13P_F	GCAGGTCGGTTCGAGCCGAGACGAGGAGCCCGGCCAA AGATGATATCAAG
6313	BCL7A_K15E_F	GCAGGTCGGTTCGAGCCGAGACGAGGAGCCGGGCCGA AGATGATATCAAGAG
6314	BCL7A_K19N_F	CAGGTCGGTTCGAGCCGAGACGAGGAGCCGGGCCAAA GATGATATCAACAGGGTCATGGC
6315	BCL7A_R20K_F	CAGGTCGGTTCGAGCCGAGACGAGGAGCCGGGCCAAA GATGATATCAAGAAGGTCATGGC
6316	BCL7A_R29C_R	CATTTGCACACTTTCTCGATCGCCG
6317	BCL7A_K30R_R	CCATCTGCGCACTTTCTCGATCGCCG

6320	BCL11A_qPCR_1F	CGCCAGAGGATGACGATTGTT
6321	BCL11A_qPCR_1R	CCAGGCGTGGGGATTAGAG
6324	ITGB2_qPCR_1F	TGCGTCCTCTCTCAGGAGTG
6325	ITGB2_qPCR_1R	GGTCCATGATGTCGTCAGCC
6328	BCL7A_R29C_F	CATGGCGGCGATCGAGAAAGTGTGCAAATGGG
6329	BCL7A_K30R_F	CGATCGAGAAAGTGCGCAGATGGGAG
6838	IFI27_qPCR_F	TGCTCTCACCTCATCAGCAGT
6839	IFI27_qPCR_R	CACAACTCCTCCAATCACAACT
6840	ITGA3_qPCR_F	TGTGGCTTGGAGTGACTGTG
6841	ITGA3_qPCR_R	TCATTGCCTCGCACGTAGC
6842	NDFIP1_qPCR_F	TTTGTGGGTCGGGATGATTTTG
6843	NDFIP1_qPCR_R	AAATGGCCCCATACCTTCCTG
6844	NRP2_qPCR_F	GCTGGCTATATCACCTCTCCC
6845	NRP2_qPCR_R	TCTCGATTTCAAAGTGAGGGTTG
6846	PECAM1_qPCR_F	AACAGTGTTGACATGAAGAGCC
6847	PECAM1_qPCR_R	TGTAAAACAGCACGTCATCCTT
6850	SMARCD3_qPCR_F	ACTGGATCAAACCATCATGCG
6851	SMARCD3_qPCR_R	CAATGCTGCCGTCGGAATC
6852	MPEG1_qPCR_F	CGGCAGCATGGGCTAAATCA
6853	MPEG1_qPCR_R	TGTCCACATTCCGCAGATTGT
6856	PLEK_qPCR_F	TTTGCCAGGAAATCTACCAGGA
6857	PLEK_qPCR_R	CGCAGTTACCTGTGAAGCAGT
6858	TTC39C_qPCR_F	CTGTTAGCTTTGGATATGGCCTT
6859	TTC39C_qPCR_R	GTCCTTACTTTCGCTTGCATACA
6860	VPREB1_qPCR_F	CGACCATGACATCGGTGTGTA
6861	VPREB1_qPCR_R	GGCTCTTGTCTGATTGTGAGAA
7573	Sink_900_F	GCGATGAAGGTGATAAATGGCGAAAC
7574	Sink_900_R	CTCCTGGACGTAGCCTTCGGGCATG
8788	Myco-F	GGGAGCAAACAGGATTAGTATCCCT
8789	Myco-R	TGCACCATCTGTCACTCTGTTAACCTC

Supplemental Table3. The list of chromatin templates used in this study

Chromatin template	Description	Product size (bp)	Plasmid Template	PCR primer 1	PCR primer 2	Restriction enzyme	Figure Number
ChT-01	216L	216	#pBL645	#6300	#6301	/	2b, 2c, 2f, 3e, S2a
ChT-02	45N45	237	#pBL386	/	/	EcoRV	2d, 3f, S4a

Supplemental Table 4. The cell lines used in this study

Cell	Name	Parental cell	Genotype	
cMD011	HEK293T	/	Wild type	Li Lab
cMD015	OCI-LY1	/	Wild type	Jiao Ma (Shen et al., 2022)
cMD018	OCI-LY10	/	Wild type	Nan Wang
cMD020	Su-DHL-4	/	Wild type	Nan Wang
cMD022	DB	/	Wild type	Nan Wang
cMD058	BAF47ΔC-t (#55-5)	cMD011	BAF47 C-terminal domain mutant	This study

Supplemental Table 5. The list of reagents or resource used in this study

Reagent or Resource	Source	Identifier
ACTB Monoclonal Antibody	Abclonal	AC004
Affinity BCL7A antibody	Affinity	DF4737
Anti V5-Tag Mouse mAb	Abclonal	AE017
BAF250 Polyclonal Antibody	Abclonal	A16648
BRD7 Polyclonal Antibody	Abclonal	A2308
BRD9 Polyclonal Antibody	Abclonal	A7133
FLAG M2 mouse monoclonal antibody	Sigma	F1804
FLAG-HRP	Sigma	A8592
Peroxidase AffiniPure Goat Anti-Mouse IgG (H+L)	AffiniPure	115-035-003
Peroxidase AffiniPure Goat Anti-Rabbit IgG (H+L)	AffiniPure	111-035-003
Rabbit IgG	Sigma	I5006
SMARCA4 Polyclonal Antibody	Abclonal	A2117
SMARCB1/BAF47 Rabbit mAb	CST	D8M1X
SMARCC1 Polyclonal Antibody	Abclonal	A6128
V5-Tag (D3H8Q) Rabbit mAb	CST	13202S
CUT&Tag kit TD903	VAZYME	TD903
ATAC-seq kit TD501	VAZYME	TD501
BiO-CZ anti-V5 affinity gel	BiO-CZ	GNI4510-V5
V5 Synthetic Peptide	SinoBiological	PP100378
Anti-DYKDDDDK Affinity Beads (Flag Beads)	Smart-Lifesciences	SA042005
Lipofectamine 3000 Transfection Reagent	Invitrogen	CW3007M
Puromycin Dihydrochloride	Gibco	A1113803
ATP solution	Sigma	GE27-2056-01
HhaI restriction enzyme	NEB	R0139S
TRIzol Reagent	Invitrogen	15596018CN
Fast RNA-seq Lib Prep Kit V2	Abclonal	RK20306
qPCR Lentivirus Titration(Titer) Kit	ABM	LV900
Lenti-X virus concentrator	Takara	631231
Matrigel Basement Membrane Matrix	Corning	354234
cBAF complex (V5-BCL7A, FLAG-DPF2)	This study	Cpmd-001
cBAF complex (V5-BCL7A-Δ20, FLAG-DPF2)	This study	Cpmd-002
cBAF complex (V5-BCL7A, FLAG-DPF2) batch#2	This study	Cpmd-004
cBAF complex (V5-BCL7A-Δ20, FLAG-DPF2) batch#2	This study	Cpmd-005
cBAF complex (BAF47ΔCt, V5-BCL7A, FLAG-DPF2)	This study	Cpmd-006
cBAF complex (BAF47ΔCt, V5-BCL7AΔ20, FLAG-DPF2)	This study	Cpmd-007
45N45-CH mono-nucleosome	This study	Cpmd-008
216L-CH mono-nucleosome	This study	Cpmd-009
hSWI/SNF core subcomplex (V5-BCL7A, FLAG-BRG1 Ct)	This study	Cpmd-015
hSWI/SNF submodule (V5-BCL7A-Δ20, FLAG-BRG1 Ct)	This study	Cpmd-016

Supplemental Table 6. The list of software and algorithms used in this study

Name	Source	Identifier
Image J	Image J (Schneider et al., 2012)	Version 1.53t
Image Lab	Bio-Rad	Version 6.0.0
CytoFLEX	Beckman	/
AlphaFold3	DeepMind (Abramson et al., 2024)	/
Bowtie2	B. Langmead et al. (Langmead and Salzberg, 2012)	Version 2.2.5
MACS2	Y. Zhang et al. (Zhang et al., 2008)	Version 2.2.6
SEACR	A. Dobin et al. (Meers et al., 2019)	Version 1.3
STAR	A. Dobin et al. (Dobin et al., 2013)	Version 2.7.10a
samtools	H. Li et al. (Li et al., 2009)	Version 1.16.1
featureCounts	Y. Liao et al. (Liao et al., 2014)	Version 2.0.1d
HOMER	S. Heinz et al. (Heinz et al., 2010)	Version 4.11
limma	M. E. Ritchie et al. (Ritchie et al., 2015)	Version 3.58.1
edgeR	M. D. Robinson et al. (Robinson et al., 2010)	Version 3.18

References:

ABRAMSON, J., ADLER, J., DUNGER, J., EVANS, R., GREEN, T., PRITZEL, A., RONNEBERGER, O., WILLMORE, L., BALLARD, A. J., BAMBRICK, J., BODENSTEIN, S. W., EVANS, D. A., HUNG, C. C., O'NEILL, M., REIMAN, D., TUNYASUVUNAKOOL, K., WU, Z., ZEMGULYTE, A., ARVANITI, E., BEATTIE, C., BERTOLLI, O., BRIDGLAND, A., CHEREPANOV, A., CONGREVE, M., COWEN-RIVERS, A. I., COWIE, A., FIGURNOV, M., FUCHS, F. B., GLADMAN, H., JAIN, R., KHAN, Y. A., LOW, C. M. R., PERLIN, K., POTAPENKO, A., SAVY, P., SINGH, S., STECULA, A., THILLAISUNDARAM, A., TONG, C., YAKNEEN, S., ZHONG, E. D., ZIELINSKI, M., ZIDEK, A., BAPST, V., KOHLI, P., JADERBERG, M., HASSABIS, D. & JUMPER, J. M. 2024. Accurate structure prediction of biomolecular interactions with AlphaFold 3. *Nature,* 630**,** 493–500.

BALIñAS-GAVIRA, C., RODRíGUEZ, M. I., ANDRADES, A., CUADROS, M., ÁLVAREZ-PéREZ, J. C., ÁLVAREZ-PRADO, Á. F., DE YéBENES, V. G., SáNCHEZ-HERNáNDEZ, S., FERNáNDEZ-VIGO, E., MUñOZ, J., MARTíN, F., RAMIRO, A. R., MARTíNEZ-CLIMENT, J. A. & MEDINA, P. P. 2020. Frequent mutations in the amino-terminal domain of BCL7A impair its tumor suppressor role in DLBCL. *Leukemia,* 34**,** 2722–2735.

DOBIN, A., DAVIS, C. A., SCHLESINGER, F., DRENKOW, J., ZALESKI, C., JHA, S., BATUT, P., CHAISSON, M. & GINGERAS, T. R. 2013. STAR: ultrafast universal RNA-seq aligner. *Bioinformatics,* 29**,** 15–21.

HEINZ, S., BENNER, C., SPANN, N., BERTOLINO, E., LIN, Y. C., LASLO, P., CHENG, J. X., MURRE, C., SINGH, H. & GLASS, C. K. 2010. Simple Combinations of Lineage-Determining Transcription Factors Prime cis-Regulatory Elements Required for Macrophage and B Cell Identities. *Molecular Cell,* 38**,** 576–589.

HUH, J.-W., WU, J., LEE, C.-H., YUN, M., GILADA, D., BRAUTIGAM, C. A. & LI, B. 2012. Multivalent di-nucleosome recognition enables the Rpd3S histone deacetylase complex to tolerate decreased H3K36 methylation levels.

The EMBO Journal, 31, 3564–3574.

LANGMEAD, B. & SALZBERG, S. L. 2012. Fast gapped-read alignment with Bowtie 2. *Nature Methods,* 9, 357–359.

LEE, C.-H., WU, J. & LI, B. 2013. Chromatin Remodelers Fine-Tune H3K36me-Directed Deacetylation of Neighbor Nucleosomes by Rpd3S. *Molecular Cell,* 52, 255–263.

LI, H., HANDSAKER, B., WYSOKER, A., FENNELL, T., RUAN, J., HOMER, N., MARTH, G., ABECASIS, G. & DURBIN, R. 2009. The Sequence Alignment/Map format and SAMtools. *Bioinformatics,* 25, 2078–2079.

LIAO, Y., SMYTH, G. K. & SHI, W. 2014. featureCounts: an efficient general purpose program for assigning sequence reads to genomic features. *Bioinformatics,* 30, 923–930.

MEERS, M. P., TENENBAUM, D. & HENIKOFF, S. 2019. Peak calling by Sparse Enrichment Analysis for CUT&RUN chromatin profiling. *Epigenetics & Chromatin,* 12.

RAMíREZ, F., RYAN, D. P., GRüNING, B., BHARDWAJ, V., KILPERT, F., RICHTER, A. S., HEYNE, S., DüNDAR, F. & MANKE, T. 2016. deepTools2: a next generation web server for deep-sequencing data analysis. *Nucleic Acids Research,* 44, W160–W165.

RITCHIE, M. E., PHIPSON, B., WU, D., HU, Y., LAW, C. W., SHI, W. & SMYTH, G. K. 2015. limma powers differential expression analyses for RNA-sequencing and microarray studies. *Nucleic Acids Research,* 43, e47–e47.

ROBINSON, M. D., MCCARTHY, D. J. & SMYTH, G. K. 2010. edgeR: a Bioconductor package for differential expression analysis of digital gene expression data. *Bioinformatics,* 26, 139–140.

SCHNEIDER, C. A., RASBAND, W. S. & ELICEIRI, K. W. 2012. NIH Image to ImageJ: 25 years of image analysis. *Nature Methods,* 9, 671–675.

SHEN, Y., ZHOU, J., NIE, K., CHENG, S., CHEN, Z., WANG, W., WEI, W., JIANG, D., PENG, Z., REN, Y., ZHANG, Y., FAN, Q., RICHARDS, K. L., QI, Y., CHENG, J., TAM, W. & MA, J. 2022. Oncogenic role of the SOX9-DHCR24-cholesterol biosynthesis axis in IGH-BCL2+ diffuse large B-cell lymphomas. *Blood,* 139 (1), 73–86.

ZHANG, Y., LIU, T., MEYER, C. A., EECKHOUTE, J., JOHNSON, D. S., BERNSTEIN, B. E., NUSBAUM, C., MYERS, R. M., BROWN, M., LI, W. & LIU, X. S. 2008. Model-based Analysis of ChIP-Seq (MACS). *Genome Biology,* 9.
